# In situ Tracking of Exoenzyme Activity Using Droplet Luminescence Concentrators for Ratiometric Detection of Bacteria

**DOI:** 10.1021/acssensors.3c01385

**Published:** 2023-11-07

**Authors:** Agata W. Baryzewska, Christian Roth, Peter H. Seeberger, Lukas Zeininger

**Affiliations:** † Department of Colloid Chemistry, Max Planck Institute of Colloids and Interfaces, Am Muehlenberg 1, 14476 Potsdam, Germany; ‡ Department of Biomolecular Systems, Max Planck Institute of Colloids and Interfaces, Am Muehlenberg 1, 14476 Potsdam, Germany

**Keywords:** emulsions, fluorescent probes, cleavable surfactants, exoenzymes, bacteria detection, foodborne pathogens, Janus droplets, responsive soft matter

## Abstract

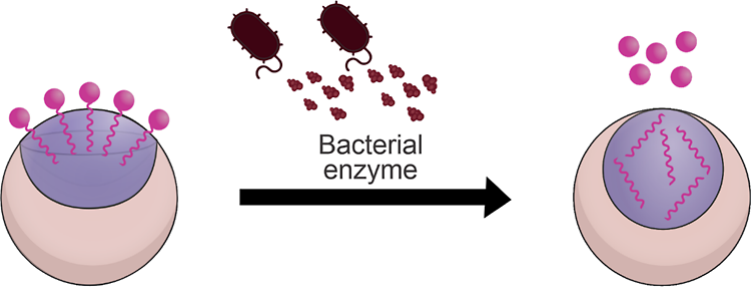

We demonstrate a novel, rapid, and cost-effective biosensing paradigm that is based on an in situ visualization of bacterial exoenzyme activity using biphasic Janus emulsion droplets. Sensitization of the droplets toward dominant extracellular enzymes of bacterial pathogens is realized via selective functionalization of one hemisphere of Janus droplets with enzyme-cleavable surfactants. Surfactant cleavage results in an interfacial tension increase at the respective droplet interface, which readily transduces into a microscopically detectable change of the internal droplet morphologies. A macroscopic fluorescence read-out of such morphological transitions is obtained via ratiometrically recording the angle-dependent anisotropic emission signatures of perylene-containing droplets from two different angles. The optical read-out method facilitates detection of marginal morphological responses of polydisperse droplet samples that can be easily produced in any environment. The performance of Janus droplets as powerful optical transducers and signal amplifiers is highlighted by rapid (<4 h) and cost-effective antibody and DNA-free identification of three major foodborne pathogens, with detection thresholds of below 10 CFU mL^–1^ for *Salmonella* and <10^2^ to 10^3^ CFU mL^–1^ for *Listeria* and *Escherichia coli*.

## Introduction

According to the World Health Organization, each year, approximately 600 million people fall ill after consuming pathogen-contaminated food.^1^ Foodborne illnesses are frequently caused by the bacteria *Salmonella enterica*, *Listeria monocytogenes*, and *Escherichia coli* that can be present in a wide variety of foods, including vegetables, dairy products, and meat, as well as drinking water.^2^ Rapidly deployable, cost-effective point-of-care sensing platforms for detecting pathogens in food are central to addressing the serious public health threat they continue to pose.^3^

Traditional bacterial culture and identification strategies require several days, which is time that is lost in preventing pathogen-contaminated food from reaching consumers. Today, ISO-certified culturing and colony enumeration methods are employed as the gold standard technology for foodborne pathogen detection.^4−6^ These methods are typically based on an enrichment and subsequent enumeration of cultures by plating samples on a selective agar medium.^7^ The method results in a high success rate and is highly cost-effective. However, depending on the pathogen, incubation times of 3–7 days required to obtain conclusive results can be fatal in preventing pathogen-contaminated food from reaching the consumer.

Advances in microbiology, analytical instruments, and sensory materials have greatly improved the speed and the detection limits for pathogen detection.^8−12^ Improved diagnostic platforms include DNA amplification and sequencing hybridization techniques, such as polymerase-chain-reaction (PCR) assays,^13,14^ antibody-based detection schemes, such as enzyme-linked immunosorbent assays (ELISA),^15^ matrix-assisted laser desorption ionization-time-of-flight (MALDI-TOF) mass spectrometric methodologies,^16^ and nanomaterial-based biosensing assays.^17,18^ Each technique has advantages and disadvantages, and its suitability for bacterial detection and identification is determined by a combination of trade-off factors, such as their specificity and sensitivity, the availability of the necessary instrumentation, and financial considerations. While some methods require bacterial pre-enrichment and incubation steps, others have reduced the read-out time to hours or even minutes but are laboratory-based, require costly equipment, or are complicated to perform. Some methods are simply too expensive for a rapid on-site testing of food products.^19^ To prevent contaminated food from reaching consumers and causing widespread disease, cost-effective, and easy-to-use sensing platforms are urgently needed. High specificity to detect miniscule amounts of pathogenic cells should be achieved ideally within a single shift in a food production plant.

Liquid–liquid transduction schemes, such as responsive Janus emulsions, are appealing because they can be easily and cost-effectively prepared from inexpensive reagents and the dynamic hydrophobic–hydrophilic liquid interfaces facilitate reactions between synthetic bioselectors and pathogens within their native aqueous environment.^20,21^ Janus emulsions consist of biphasic emulsion droplets, such as phase-separated mixtures of hydrocarbon (HC) and fluorocarbon (FC) oils, dispersed within an aqueous surfactant-containing continuous phase.^22^ The force balance of interfacial tensions acting at the individual interfaces dictates the internal geometry of Janus droplets.^23,24^ Therefore, Janus droplets are intrinsically responsive to changes in their chemical environment.^25^ Marginal changes in the interfacial tension balance, triggered by altering surfactant concentrations or effectiveness, transduce into immediate changes in the internal droplet morphology.^26,27^ Microscale changes in droplet morphology, result in a significant modulation of their macro-scale optical characteristics.^28,29^ Underpinned by the tunable refractive index contrast of the constituent fluids of the droplets combined with their gravitational alignment, Janus emulsions represent a versatile material platform for manipulating the pathway of light passing through these microscale elements. Besides leveraging this unique morphological-optical coupling in dynamic refractive, reflective, and light-emitting optical components,^30^ solar concentrators,^31^ or for structural coloration,^32^ Janus emulsions offer rich opportunities as modular sensing layers that enable optical transduction and signal amplification in liquid sensing platforms.^33−36^

Two different responsive modalities of Janus emulsions have been used to convert biochemical recognition events to a readable signal. Either multivalent supramolecular or competitive dynamic covalent binding events that emulate biological detection strategies such as protein–protein or carbohydrate–protein interactions were used to evoke changes in droplet orientation,^37,38^ or variations to the internal droplet geometry.^39,40^ Sensitivities for the detection of bacteria ranged from 10^3^ to 10^5^ cells mL^–1^ in these pilot studies, which rendered them competitive with commercial detection methods. We hypothesized that an alternative chemical-morphological signal transduction pathway, where covalent changes in surfactant composition are induced by enzymatic cleavage, would result in pronounced variations in the interfacial tension balance. In addition, innovations in the morphological-optical signal transduction provide the basis for an increase in sensitivity and improvement in the signal-to-noise ratio, thus unlocking the potential for the creation of more robust Janus emulsion-based sensing schemes for a rapid early-stage detection of a broad range of foodborne pathogens.

We herein demonstrate a new Janus emulsion-based sensing scheme that enables rapid detection and discrimination between the three major foodborne pathogens, *S. enterica*, *L. monocytogenes*, and *E. coli*. Our detection strategy is founded on the visualization of bacterial exoenzyme activity via morphological changes of Janus droplets, initiated by enzyme-catalyzed cleavage of Janus emulsion surfactants. We employed three different target surfactants that served as selective substrates for the most dominant extracellular hydrolase enzymes of the respective bacteria strains, namely, C8-esterase, β-glucosidase, and β-galactosidase. Enzyme-mediated cleavage of the surfactants’ hydrophilic head groups transduced into immediate changes in the emulsion droplets’ internal morphologies due to the reconfigurable nature of Janus emulsions. Morphological transitions were monitored in situ, both microscopically and by a new macroscopic fluorescence-based read-out technique. Precise quantification of marginal morphological transitions was realized via tracking unique anisotropic light emission characteristics of dyed Janus emulsions by implementing a dual-angle-dependent ratiometric detection strategy.

## Results and Discussion

Living bacteria cells continuously produce extracellular enzymes that they release to the environment.^41^ Certain exoenzymes are characteristic to specific bacteria, and thus allow for differentiation between different bacterial species.^42−44^ A commonly used method for bacterial identification in microbiology laboratories involves the use of chromogenic media. These media require inoculation of small amounts of clinical specimens from suspected infection sites using optimal broth or agar media in the presence of the chromogenic or fluorogenic substrates, which stain in the presence of specific enzymes expressed by the bacterium of interest. The underlying mechanism involves an enzymatically catalyzed hydrolysis of a colored or fluorescent marker, such as 4-methylumbelliferon. We anticipated that coupling this bacterial-specific cleavage mechanism with a responsive emulsion platform would facilitate the use of such synthetic molecular sensors with limited solubility under native aqueous biosensing conditions to enable a direct in situ visualization of exoenzyme-mediated hydrolysis. This would aid in the development of a rapid and cost-effective on-site detection method to identify food samples contaminated with pathogens at levels less than 1 CFU per 25 g, that meet the limit of detection thresholds required by the FDA and EU regulations.^45,46^

**Figure 1:**
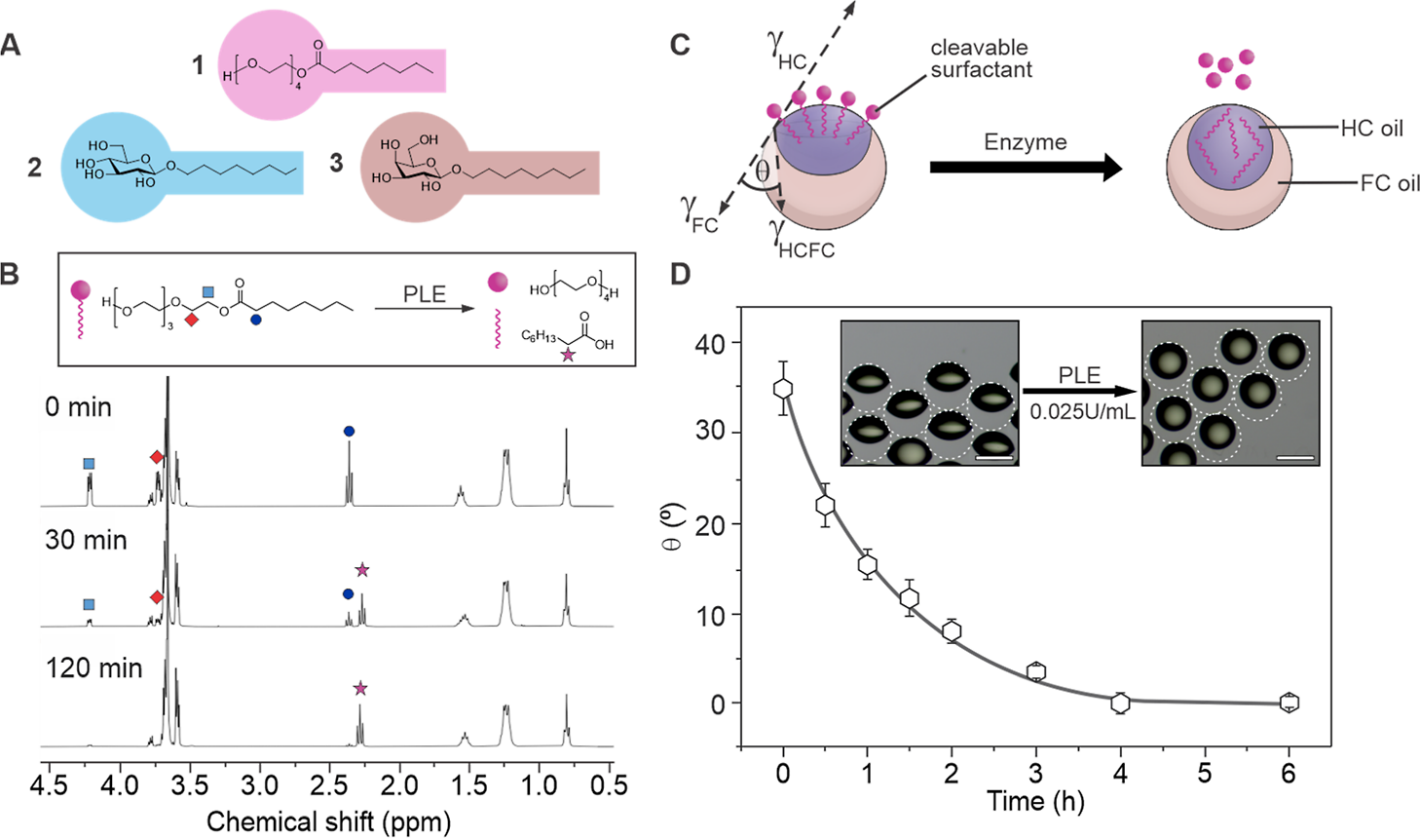
Enzyme-mediated cleavage of the target surfactants. (A) Chemical structures of the cleavable surfactants tetra(ethylene glycol)mono-*n*-octanoate (**1**), β-*n*-octyl glucopyranoside (**2**), and β-*n*-octyl galactopyranoside (**3**) used in this study; (B) time-dependent ^1^H NMR spectra for the enzymatic cleavage of **1** by PLE (1 U mL^–1^); (C) schematic illustration of the Janus droplet-based visualization of enzymatic activity via morphological transitions induced by the cleavage of target surfactants; (D) time-dependent morphological transitions of Janus droplets stabilized by surfactant **1** upon addition of C-8 porcine liver esterase (PLE) (0.025 U mL^–1^). Inset micrographs display starting and end morphologies of Janus droplets comprised of diethylbenzene and HFE7500; error bars denote *N* ≥ 5 measurements; scale bar: 100 μm.

To sensitize Janus droplet transducers toward a detection of the foodborne pathogenic bacteria *S. enterica*, *L. monocytogenes*, and *E. coli*, we therefore employed surfactants targeting the predominant extracellular enzymes, namely C8-esterase,^47^ β-glucosidase,^48^ and β-galactosidase,^49^ respectively. The specific substrates, tetra(ethylene glycol)mono-*n*-octanoate (**1**), β-*n*-octyl glucopyranoside (**2**), and β-*n*-octyl galactopyranoside (**3**) (Figure 1A), possess an intrinsic amphiphilicity due to the hydrophilic polyethylene glycol or carbohydrate head groups, which were attached to hydrophobic alkyl chains. These molecules successfully stabilized oil-in-water emulsions, and their surface activity was confirmed by pendant drop tensiometry. The interfacial tension between a hydrocarbon oil (diethylbenzene) and water was reduced to 4.9 mN m^–1^ for **1**, 1.3 mN m^–1^ for **2,** and 1.5 mN m^–1^ for **3**, above the surfactants’ respective critical micelle concentrations of 10.0 mM for **1**,^50^ 28.3 mM for **2**,^51^ and 31.5 mM for **3**.^51^

To confirm the surfactants’ function as selectors in our biosensing strategy, we investigated the enzyme-mediated cleavage of the amphiphiles using ^1^H NMR spectroscopy (Figures 1B and S7–S9). We observed a time and enzyme concentration dependence of the cleavage of the synthetic molecular surfactants, which was analogous to commercial chromogenic or fluorogenic substrates (Figure S10),^52^ with longer incubation times and higher enzyme concentrations resulting in pronounced surfactant cleavage. Upon incubation of surfactant **1** (0.1 wt %) with 1 U mL^–1^ of C8 porcine liver esterase (PLE) in D_2_O, a time-dependent recording of the ^1^H NMR spectrum showed full cleavage of the surfactant within 2 h. Time-dependent recording of the ^1^H NMR spectra of surfactants **2** and **3** similarly showed a gradual cleavage of the surfactants over time upon incubation with the respective enzymes.

To investigate the specificity of enzymatic cleavage, a series of cross-tests were performed in which each surfactant was incubated with enzymes other than the target (Figures S11–S16). The cross-tests revealed no or negligible cleavage of the molecules after four hours of incubation, demonstrating the high specificity of the detection strategy.

As opposed to single-phase emulsion droplets, where changes in surfactant effectiveness and thus variations in interfacial tensions result in only qualitative results, such as changes in droplet size or stability, Janus emulsions possess the intrinsic advantage that interfacial tension variations transduce into changes in the internal droplet geometry, whereas the overall emulsion stability remains intact. To generate Janus emulsions comprised of a 1:1 volume mixture of the hydrocarbon oil diethylbenzene and the fluorocarbon oil HFE-7500 as the droplet constituent phases, we employed an established thermal phase separation approach.^23^ The generated droplets were polydisperse in size; however, presented a highly uniform internal morphology, i.e., uniform internal shape and volume ratio of the constituent droplet phases across a sample. While emulsions stabilized exclusively by the surfactants **1**, **2**, or **3** adopted an encapsulated double emulsion morphology with the hydrocarbon encapsulating the fluorocarbon, stabilization of the droplets employing a mixture of the HC with a FC surfactant (Zonyl FS-300; hereafter: Zonyl) resulted in droplets adopting a Janus geometry (Figure 1C). In these droplets, the constituent fluids aligned by gravity, which placed the denser FC-phase at the bottom.

To visualize changes in Janus droplet morphology due to an enzymatic cleavage of the three target surfactants, we employed a customized microscopic side-view imaging setup. The respective enzymes were added to the aqueous emulsion continuous phase containing a mixture of surfactant **1**, **2**, or **3** with Zonyl (0.1 wt %). The concentration of the surfactants was adjusted to ensure identical starting Janus morphologies of the droplets. Changes in droplet morphology were then quantified via the determination of changes in the triple-phase contact angle (θ) (Figure 1D).

When microscopically monitoring Janus droplets stabilized by surfactant **1** (9.3 mM) after the addition of PLE (0.025 U mL^–1^) over time, we observed a morphological transition from the “opened-up” Janus (θ = 35°) toward an encapsulated double emulsion (θ = 0°) morphology within *t* = 4 h. This could be attributed to the preferred assembly of the cleavable surfactants at the hydrocarbon–water interface of the droplets, and hence, a cleavage-induced gradual increase of the interfacial tension at the latter. Analogously, enzyme cleavage kinetics were tracked using droplets stabilized by surfactant **2** (10.2 mM) or **3** (13.6 mM). A transition into a fully encapsulated double emulsion morphology within 4 h was observed upon addition of β-glucosidase (1.0 U mL^–1^) and β-galactosidase (2.5 U mL^–1^), respectively (Figures S17 and S18). The necessity for higher concentrations of both carbohydrate hydrolase enzymes reflected their decreased activities in the PBS-buffered emulsion continuous phase (pH 7.4), which, however, was chosen to ensure a physiological pH within the following bacteria sensing experiments.^53^

**Figure 2:**
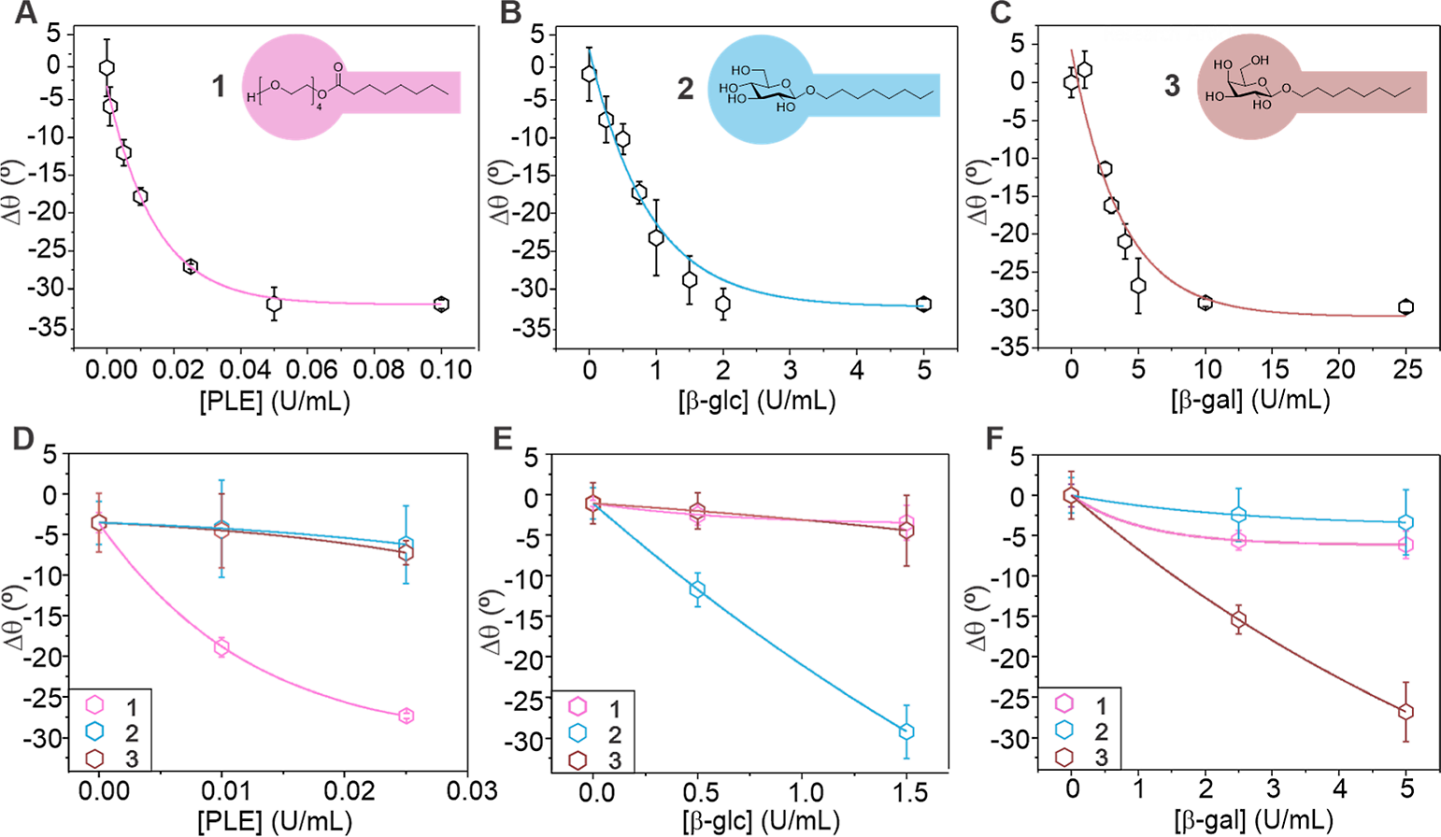
In situ visualization of enzyme activity via morphological responses of Janus emulsion droplets. (A–C) Changes in Janus emulsion morphology quantified via the triple-phase contact angle (θ) of droplets functionalized with surfactants **1** (A), **2** (B), or **3** (C) as a function of the respective target enzyme concentration as determined from side-view micrographs of the droplets after 2 h; (D–F) Cross-tests showed only marginal responses of droplets sensitized with surfactant **1**, **2**, or **3** toward enzymes other than the target enzyme; error bars denote *N* ≥ 5 measurements.

Next, we investigated the change in the droplet morphology at different enzyme concentrations (Figure 2A–C). The calibration curves for the enzyme concentration dependence of the cleavage of surfactants **1**, **2**, and **3** after 2 h show that at low concentrations, the Janus droplet contact angle decrease and enzyme concentration are linearly correlated and thus provide a precise approach to monitor enzymatic activity of PLE (*R*^2^ = 0.96), β-glucosidase (*R*^2^ = 0.95), and β-galactosidase (*R*^2^ = 0.96) (Figures S25–S27). Cleavage of ester surfactant **1** by C8-esterase occurred more quickly, likely due to the more favorable pH conditions. Overall, the sensing strategy allowed for the detection of low enzyme concentrations with readily detectable morphology changes after two h induced by 0.005 U mL^–1^ PLE, 0.5 U mL^–1^ β-glucosidase, and 2.5 U mL^–1^ β-galactosidase, respectively. Cross-tests revealed a good specificity of the respective Janus droplet probes that were functionalized with either surfactant **1**, **2**, or **3** toward their target enzymes (Figure 2D–F). At enzyme concentrations within the sensitivity range of the respective sensing scheme, only marginal changes in droplet morphology were observed when incubated with the other two enzymes, and only droplets sensitized with the respective target surfactant produced a significant response.

**Figure 3:**
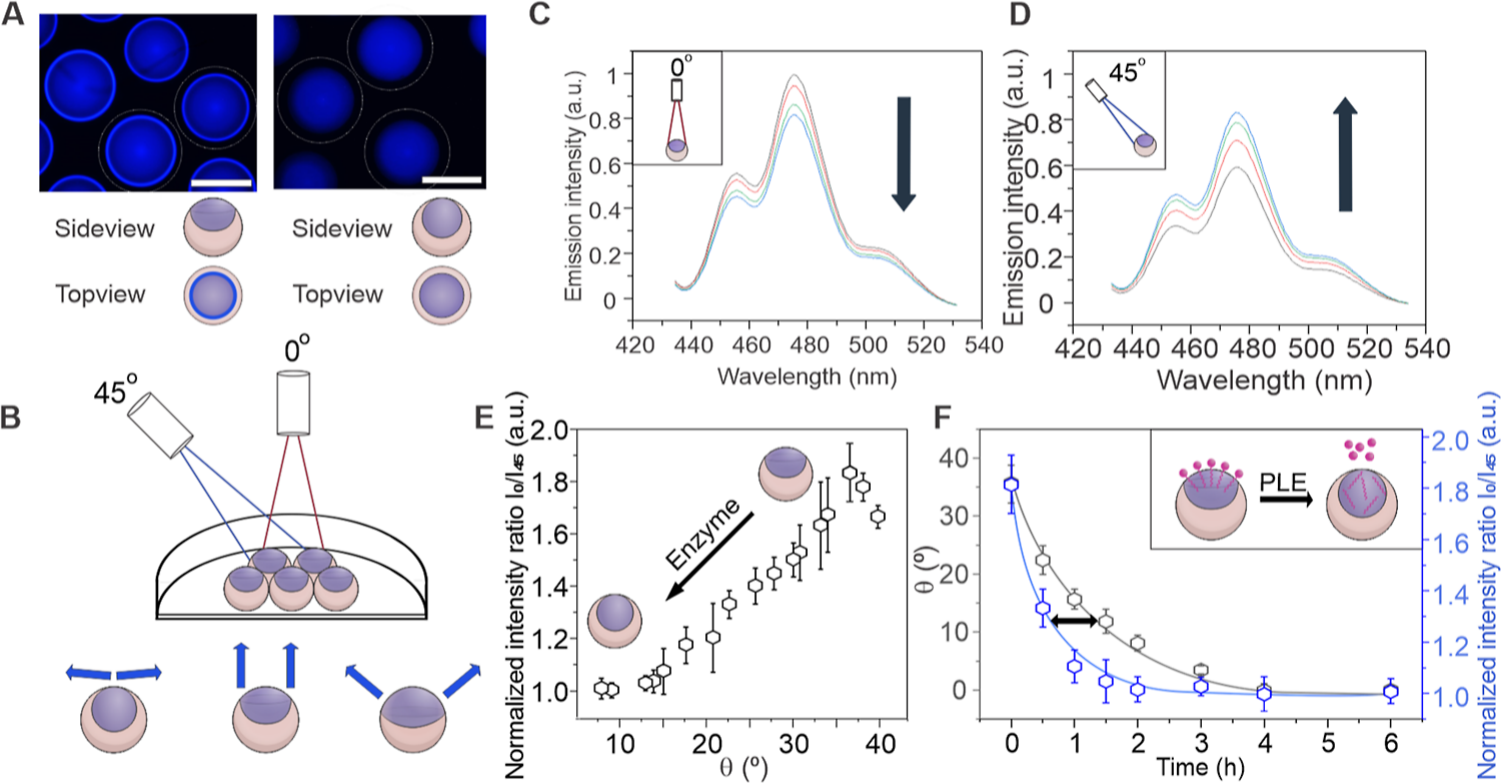
Ratiometric detection of marginal morphological responses of Janus emulsions via morphology-dependent angular anisotropic emission signatures. (A) Top-down fluorescence micrographs of Janus droplets containing 2.5 mM perylene dispersed within the HC phase in two morphologies (θ = 35° and θ = 0°); scale bar: 100 μm; (B) schematic representation of the ratiometric angle-dependent fluorescence intensity detection setup; (C,D) perylene emission intensity variations recorded upon morphological transition of Janus droplets (θ = 35°) toward the encapsulated double emulsion state (θ = 0°). A gradual decrease in the recorded light intensity recorded by the vertical probe (C) results in an opposite response in the 45° probe (D); (E) ratiometric L-curve: droplet morphology dependent emission intensity ratio collected by the two probes in 0 and 45° direction above a monolayer of polydisperse droplets; (F) sensitivity comparison between microscopic side-view contact angle determination and the fluorescence detection scheme for the visualization of PLE activity using Janus droplets stabilized with surfactant **1**.

Reliable transduction of the biochemically triggered alterations in complex droplet morphology into readable and quantifiable physical signal output is central to the development of a new biosensing platform. In Janus droplets, variations in the chemical droplet environment that lead to microscale changes in the droplet geometry can cause macroscale variations in their optical properties.^28^ Leveraging this unique chemical-morphological-optical coupling inside Janus emulsion droplets, we next set out to convert morphological transitions triggered by an enzymatic cleavage of Janus droplet surfactants into an easily detectable fluorescent readout signal. Adding perylene emitters (2.5 mM) to the droplets resulted in selective partitioning into the optically denser hydrocarbon phase, which resulted in optical confinement of the perylene emission. Due to total internal reflection trajectories of light emitted from inside the droplets, Janus emulsions display a morphology-dependent angular anisotropic emission signature, as previously revealed both experimentally and theoretically through ray tracing models.^20,54,55^ Vertically monitoring the fluorescence output of gravitationally aligned “opened-up” Janus droplets in a morphology corresponding to the starting point of our sensing scheme using a fluorescence microscope revealed a ring of concentrated emission adjacent to the triple-phase contact line, which corresponds to the region where TIR light is collected (Figure 3A). This observation is in stark contrast to droplets in a closed-up encapsulated double emulsion morphology, where no intensity increase was observed in the vertical direction. Droplets in fluorocarbon-dominant Janus morphologies, i.e., droplets with low contact angles, exhibited an increased emission output in the 45° direction as a result of changes in the out-coupling angle of the TIR collected light (Figure 3B). Based on this morphology-dependent angular anisotropic emission intensity signature, a novel dual-angle-dependent ratiometric fluorescence read-out facilitated a precise determination of marginal changes in droplet morphology. Two collection fibers were placed in 0 and 45° angles above gravity-aligned droplet monolayers. A decrease in droplet contact angle from the starting morphology (θ = 35°) toward more encapsulated morphologies (→ θ = 0°) was followed by a decrease of the collected light intensity by the vertical (0°) fiber as well as a simultaneous increase in light intensity collected by the 45° fiber (Figure 3C,D).

Ratiometric determination of the emission intensities collected by the two fibers resulted in a unique emission intensity signature of droplets in different morphologies, in the following termed as the ratiometric light-curve (L-curve). The L-curve shows a decrease in the emission intensity ratio within the range of interest for our sensing scheme. Capturing the light emitted by the droplet monolayers from two distinctly different angular ranges allowed the accurate detection of small variations in droplet morphology and the generation of ratiometric intensity calibration curves for droplets with different morphologies. This optimization of experimental conditions allowed fine-tuning of the sensitivity of the fluorescent droplet morphology to subtle changes in its chemical environment, with maximum optical responses within the contact angle range of interest between θ = 35° and θ = 15°.

Contact angle variations of Δθ < 20° visualize variations in the balance of interfacial tensions on the order of Δ(γ_HC_ – γ_FC_) 0.5 mM m^–1^,^24^ which highlights the sensitivity of the method. Recording the time-dependent detection of PLE activity yielded maximum signal changes within one h, compared to 2 h for microscopically monitoring droplet contact angle variations (Figure 3F). With droplet sizes significantly larger than the wavelength of light, the angular anisotropic emission signature was independent of the droplet sizes and provided a readout method suitable for polydisperse droplet monolayers that can be easily produced in any environment. The latter is important as it enables the application as sensors with simple on-site batch droplet generation, avoiding the need for tedious microfluidic generation of monodisperse droplet samples and complicated equipment.

To test this hypothesis, we employed our novel fluorescence-based droplet morphology read-out technique for detecting the prominent foodborne pathogenic bacteria *S. enterica*, *E. coli,* and *L. monocytogenes*. To this end, different concentrations of bacteria were added to polydisperse Janus emulsions functionalized with surfactants **1**, **2**, or **3**. Due to the differences in exoenzyme composition within each bacteria sample, we observed different responses of the droplets.

**Figure 4:**
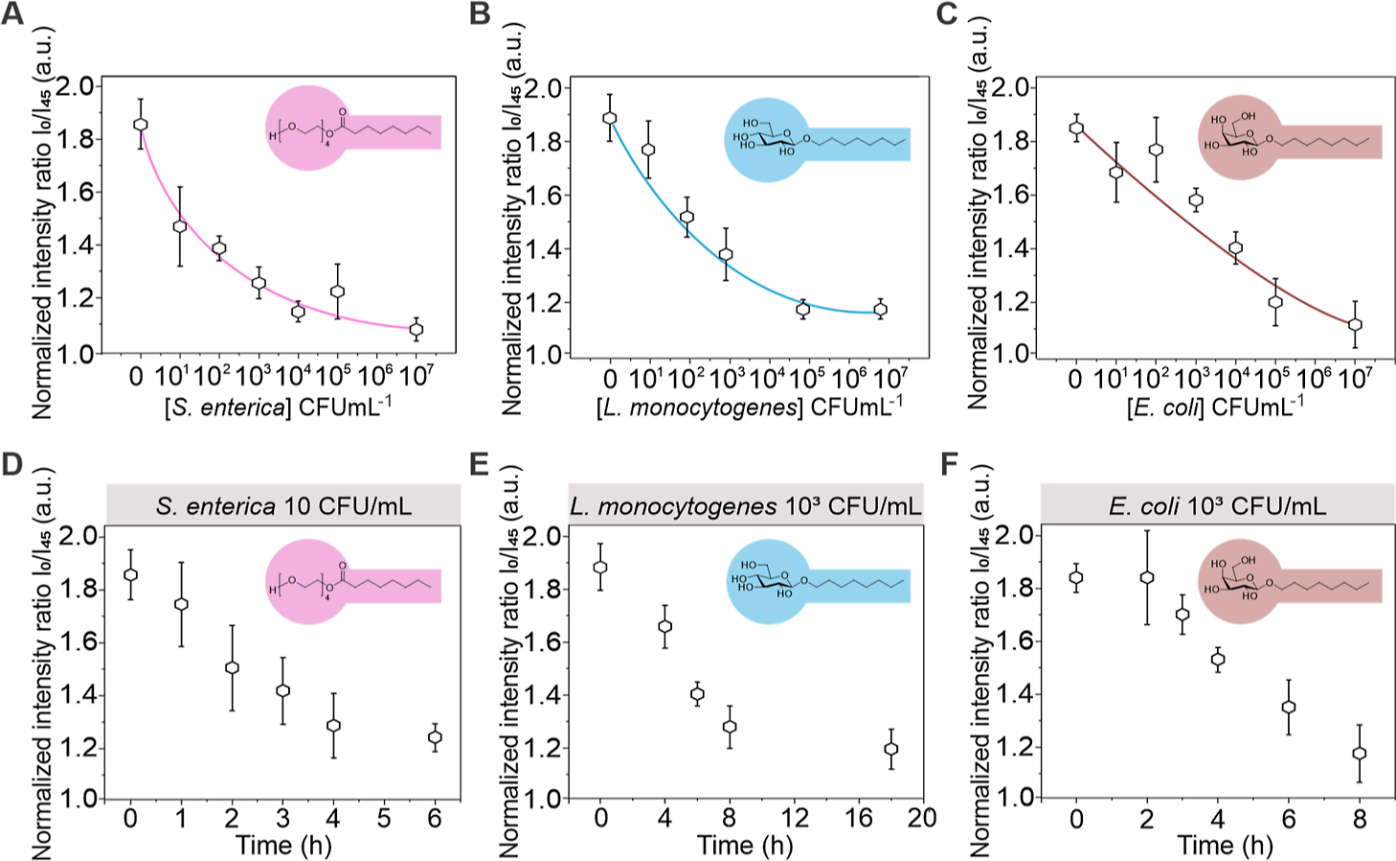
Detection of *S. enterica*, *E. coli,* and *L. monocytogenes* using droplet luminescence concentrators. (A–C) Measured ratiometric emission intensities and standard deviations (*N* ≥ 5) of droplets functionalized with surfactants **1**, **2**, or **3**, as a function of the target bacteria concentrations, recorded after 2 (A), 6 (B), and 4 h (C); (D–F) time dependency of the detection of *S. enterica*, *E. coli,* and *L. monocytogenes*; error bars denote *N* ≥ 5 independent measurements.

Selective cleavage of the respective Janus emulsion surfactants was followed via progression along the fluorescence L-curve, starting from droplets that exhibited the highest ratiometric emission intensities. Concentration-dependent monitoring of the droplets’ ratiometric emission intensity revealed detection limits exceeding previously reported Janus emulsion-based biosensing paradigms by orders of magnitude (Figure 4). The detection limit of droplets functionalized with surfactant **1** toward *S. enterica* showed that concentrations of 10 CFU mL^–1^ led to a pronounced and conveniently detectable ratiometric emission intensity signal change of ∼−50% after 2 h (Figure 4D). Single-pathogen (1 CFU mL^–1^) detection experiments (Figure S31) revealed slowed surfactant cleavage due to the lowered exoenzyme concentration within the sample but yielded a reliable positive sensor result after a 6 h incubation time. Tracking the ratiometric emission intensity change of droplet samples sensitized with surfactants **2** and **3** targeted the most prominent exoenzymes of *L. monocytogenes* and *E. coli*. Due to the decrease in enzymatic activity at physiological pH, incubation times were extended to 6 and 4 h, respectively. For the detection of *L. monocytogenes* (Figure 4B) and *E. coli* (Figure 4C), theoretical limits of detection (T-LOD) values of 5.8 and 42.8 CFU mL^–1^ were determined. Pronounced changes in droplet morphology, resulting in a >50% change in ratiometric emission intensity within the time frame, were determined upon addition of 10^2^ CFU mL^–1^ for *L. monocytogenes* and 10^3^ CFU mL^–1^ for *E. coli*.

Time-dependent monitoring of the emission intensity at these bacteria concentrations (Figure 4D–F) revealed that pronounced changes in emission intensity were obtained by prolonging the surfactant incubation time, with maximum changes reached after 4 h for *S. enterica*, and 8 h for both, *L. monocytogenes* and *E. coli* detection.

Higher standard deviations in these time-dependent studies are likely due to variations in the final concentration of the target enzymes. Whereas droplet morphology is highly uniform across a sample (Δθ < 3°), and experiments with pure and defined enzyme concentrations resulted in low standard deviations of Δθ ∼ 5°, the intrinsic recognition paradigm for bacteria sensing relies on the living organism’s metabolism to produce exoenzymes that catalyze the surfactant cleavage. Moreover, additional nontargeted interactions of the surfactants with other proteins of the bacteria cannot be excluded.

Financial considerations play a central role in the development of platforms for rapid on-site food monitoring. In addition to the specificity and sensitivity of a method, its detection speed and the availability of the necessary instruments, its cost-effectiveness is a central requirement for its applicability and feasibility in the context of detecting foodborne pathogens.^56^ Consequently, despite the availability of nucleic acid and immunological-based methods that can identify different bacteria strains and viruses with high certainty and extremely low detection thresholds, the majority of food tests to date are performed using culture-based means of identification, despite the associated time delay in obtaining a positive result.^10,11^ Consideration of this need for simplicity highlights that sensor layers or arrays based on Janus emulsions hold promise to address an unmet need for the development of rapid on-site screening platforms for monitoring food safety that are complementary to existing, more accurate but complicated methods. DNA- and antibody-free droplet sensors made from minimal starting materials could be produced in any environment and at extremely low cost, and the unique morphological-optical coupling inside Janus droplets allows facile optical transduction of chemical information in situ that can be easily multiplexed, thereby alleviating the need for complicated read-out instrumentation.

**Figure 5:**
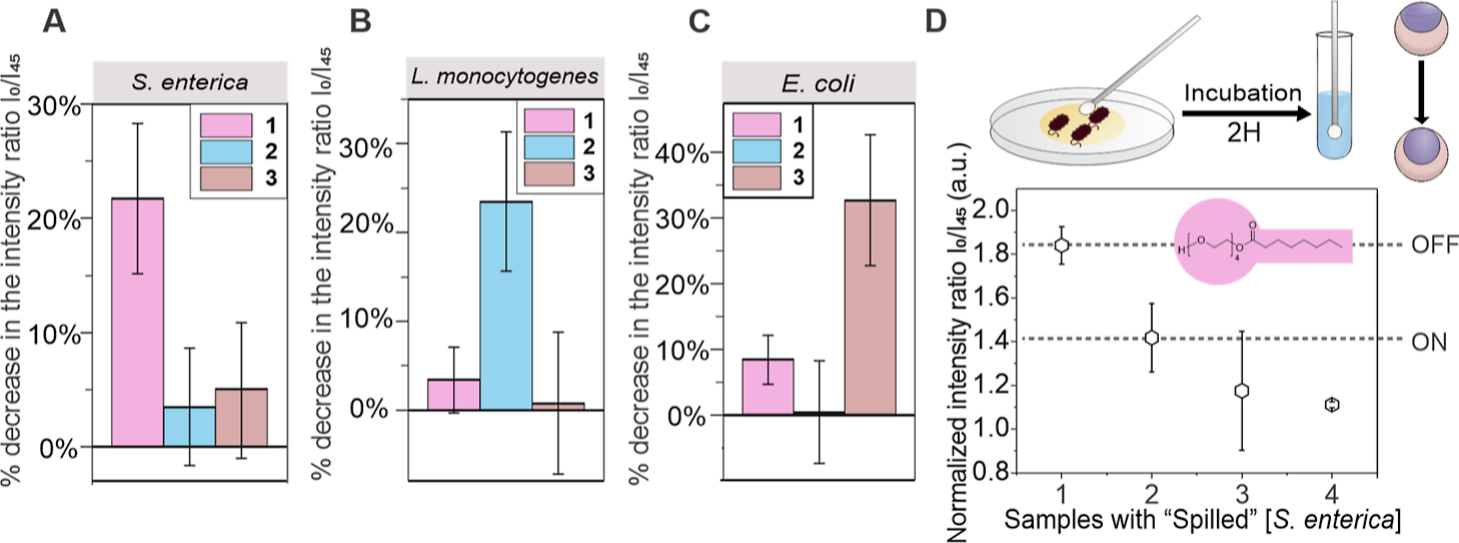
Specificity of the detection strategy and swab-based analysis of bacteria-contaminated substrates. (A–C) Measured ratiometric emission intensities of Janus droplet samples, functionalized with surfactants 1, 2, or 3 upon incubation with *S. enterica* (10^2^ CFU mL^–1^) (A) *L. monocytogenes* (10^3^ CFU mL^–1^) (B) and *E. coli* (10^4^ CFU mL^–1^); (C) error bars denote ≥5 measurements; (D) swab test results for the detection of “spilled” *S. enterica* solutions; the “spilled” sample concentrations were 0 for sample 1, 10^3^ CFU mL^–1^ for sample 2, 10^4^ CFU mL^–1^ for sample 3, and 10^7^ CFU mL^–1^ sample 4; error bars denote ≥3 independent results.

To showcase this modularity of optically active Janus droplet sensor layers, we performed experiments, where we exposed an array of differently sensitized droplet probes to bacterial solutions other than the target bacteria. In these proof-of-concept tests, it was possible to successfully discriminate between the different samples depending on the selective presence of the exoenzymes of the target bacteria. The response of emulsions functionalized with nontarget surfactants was significantly reduced compared to the target surfactants in these cross-tests (Figure 5A–C).

Motivated by these results, we next carried out swab-based blind tests for the detection of *S. enterica*, *L. monocytogenes*, and *E. coli* (Figures 5D and S32 and S33). Bacteria-containing samples were first plated on glass substrates, and a small fraction of the spilled sample was subsequently wiped with a swab. The swab was then rinsed with 1 mL of the bacteria-appropriate growth medium and incubated for 2 h at 37 °C prior to addition of the emulsion probes. A ratiometric fluorescence-based read-out of changes in the droplet contact angles then revealed pronounced changes for samples containing the target bacteria, whereas the signal from non-contaminated samples remained unchanged.

## Conclusions

In summary, we describe a novel sensitive and cost-effective biosensing signal transduction and amplification strategy that is based on an in situ visualization of bacterial exoenzyme activity using dyed Janus emulsions. We sensitized biphasic Janus droplets toward the most prominent extracellular enzymes of the common bacterial food pathogens *S. enterica*, *L. monocytogenes*, and *E. coli* via interface-selective functionalization with enzyme-cleavable surfactants. Surfactant cleavage resulted in an immediate increase in the interfacial tension at the respective droplet interface, which was followed by a morphological reconfiguration of the internal droplet geometry. An associated change of the angular-dependent anisotropic emission intensity signature of dyed droplets was then used to monitor marginal variations in morphology via a dual-angle-dependent ratiometric fluorescence signal. We showcased that a sensitive and specific detection of the bacteria *S. enterica*, *L. monocytogenes*, and *E. coli* is possible with exoenzyme type and concentration-dependent detection limits competitive with current methods of detecting and identifying pathogenic bacteria. Within for rapid on-site detection desirable time scale of 4 h, the sensing paradigm successfully identified bacteria concentrations of less than 10 CFU mL^–1^ for *S. enterica* and <10^2^ to 10^3^ CFU mL^–1^ for *L. monocytogenes* and *E. coli*.

While our demonstrations showed proof of concept and met benchmarked target ranges and general sensitivity requirements for the detection of a number of bacterial strains from food samples, additional challenges exist for a translation of this sensing paradigm into on-site, rapid food screening devices for analyzing complex food matrices. Broad adoption of the transduction scheme will require the identification of specific bacterial strains also within complex mixtures, and future efforts will be directed toward tracking multiple independent (bio)chemical interactions simultaneously. In addition, we aim to expand our morphological-to-optical read-out paradigm to function also in opaque and light-absorbing analyte media. However, the unprecedented cost-effectiveness of the overall sensing scheme, the sensitivity toward live bacteria cells, combined with the fact that the presented detection scheme can be readily miniaturized and the polydisperse emulsions are easily and cheaply prepared in any environment, holds promise to provide as a new rational alternative for randomized, on-site premonitoring of pathogen contamination in food samples that is complementary to existing methods. We further anticipate that this platform technology will also be useful for the development of diagnostic probes targeting other exoenzymes of pathologically relevant bacteria, as well as for an in situ real-time monitoring of bacterial growth.

## Supporting Information Available

Materials and methods; synthesis and characterization of enzyme-cleavable surfactants; characterization of enzyme cleavage kinetics; and detailed bacteria sensing procedures (PDF)
