# Supplementary material for: In situ Tracking of Exoenzyme Activity Using Droplet Luminescence Concentrators for Ratiometric Detection of Bacteria
Source: ACS Sens. 2023 Nov 7;8(11):4143–51. doi: 10.1021/acssensors.3c01385 (PMC10683504; doi:10.1021/acssensors.3c01385)
Supplement: Supplementary file 1 — se3c01385_si_001.pdf [file se3c01385_si_001.pdf]

SUPPORTING INFORMATION

In-situ Tracking of Exoenzyme Activity using Droplet Luminescence Concentrators for Ratiometric Detection of Bacteria

Agata W. Baryzewska,† Christian Roth,‡,* Peter H. Seeberger,‡ and Lukas Zeininger†,*

† Department of Colloid Chemistry, Max Planck Institute of Colloids and Interfaces, Am Muehlenberg 1, 14476 Potsdam, Germany; *corresponding author e-mail: lukas.zeininger@mpikg.mpg.de

‡ Department of Biomolecular Systems, Max Planck Institute of Colloids and Interfaces, Am Muehlenberg 1, 14476 Potsdam, Germany

Table of Contents

1. Materials and methods **3**

1.1 Chemicals 3

1.2 Instruments 3

1.3 Droplet preparation 4

1.4 Imaging and Microscopy 4

1.5 Contact angle analysis 4

1.6 Ratiometric angle-dependent emission detection setup 5

1.7 Bacteria culture 5

2. NMR spectra **8**

2.1 ^{1}H and ^{13}C NMR spectra of synthesized surfactants **1-3** 8

2.2 ^{1}H NMR cleavage studies – time dependency 12

2.3 ^{1}H NMR cleavage studies - enzymatic cross-tests 15

3. Detection of enzymatic activity using Janus double emulsions **19**

3.1 Determination of the specificity of the system – enzymatic cross tests 21

3.2 Enzymatic cleavage in Janus emulsions - time dependence studies 23

3.3 Enzyme kinetics studies 24

3.2 Enzymatic cleavage in Janus emulsions 28

4. Bacteria sensing **30**

4.1 General procedure for bacteria sensing with Janus emulsions 30

4.2 Detection of 1 CFU/mL of *S. enterica* – time dependence study 30

4.3 Concentration dependence and theoretical limit of detection studies 31

4.4 Cross tests 33

4.5 Swab tests 35

1. Materials and Methods:

1.1 Chemicals:

All chemicals listed were used as received without further purification: Dulbecco's phosphate buffered saline (Sigma Aldrich), diethylbenzene (95%, Sigma Aldrich), Zonyl-FS300 (40% solid in water, abcr GmbH), HFE-7500 (99%, abcr GmbH), Perylene (Sigma Aldrich), β-glucosidase from almonds (lyophilized powder, Sigma Aldrich) , β-galactosidase from *Aspergillus oryzae* (Sigma Aldrich), porcine liver esterase (lyophilized powder, Sigma Aldrich), D-(+)-glucose (Karl Roth), D-(+)-galactose (Sigma Aldrich), tetraethylene glycol (for synthesis, Sigma Aldrich), octanoyl chloride (99%, Sigma Aldrich), diethyl ether (Sigma Aldrich), dichloromethane (Sigma Aldrich), acetyl chloride (98%, Sigma Aldrich), pyridine (99%, Sigma Aldrich), ethyl acetate (VWR) , methanol (VWR), hexane (Sigma Aldrich), 1-octanol (for synthesis, Sigma Aldrich), Sodium methoxide (0.5M solution in methanol, Sigma Aldrich), deuterium oxide (Sigma Aldrich), deuterated methanol (Sigma Aldrich), tryptic soy broth (Sigma Aldrich), brain heart infusion (Sigma Aldrich), lysogeny broth (Roth). The surfactants **1-3** were synthesized according to procedures previously reported in the literature.[1,2] Surfactants **2** and **3** were additionally purchased from Sigma Aldrich and used interchangeably with synthesized surfactants **2** and **3**.

1.2 Instruments:

Nuclear magnetic resonance spectra were carried out using a Bruker Advance (400MHz) spectrometer. The angle-dependent droplet emission was recorded using an Avantes (model*: StarLine AvaSpec-ULS2048CL-EVO-RS*) spectrometer. Interfacial tension measurements were carried out using a drop shape ananalyzer tensiometer (DSA10-MK2, Krüss) in the pendant drop setting and images were recorded using a CCD camera. Bacteria samples were incubated using incubator from Binder (model BD 23) set to 37°C.

1.3 Droplet preparation:

Janus droplets comprising a 1:1 volume ratio of diethylbenzene (containing 2.5mM perylene) and HFE7500 were prepared by batch one-step emulsification via an established thermal phase-separation approach.[3] Emulsion droplets prepared by batch-scale vortex mixing were polydisperse in size displaying an average diameter of 55.7 µm ± 51.9 µm. In brief, the oil

mixture and the surfactant-containing aqueous continuous phase were separately heated above the critical solution temperature of the two oils. Subsequently, 10 vol.% of the homogenous oil mixture were transferred to the aqueous solution and vortexed at 2100 rppm for 10 sec Vortex Genie 2, Scientific Industries). After emulsification, single phase droplets were allowed to settle and cool back to room temperature, which induced phase separation of the two oils inside the droplets. The morphology of droplets is exclusively determined by the force balance of interfacial tensions acting at the individual interfaces that can be fine-tuned and controlled by adjusting the surfactant composition in the continuous phase. Emulsion droplets are kinetically stabilized and are therefore prone to aging and mechanical agitation. In our experiments we therefore employed droplets freshly prepared on the same day. However, we did not observe any noticeable changes in droplet morphology, composition, or size distribution when stored on the benchtop for up to 7 days.

1.4 Imaging and microscopy:

Side-view micrographs of Janus droplets were recorded with a BRESSER MicroCam SP 3.1 microscope camera (and MicroCamLabII software), using a custom-made side-view microscopy setup, comprising of a 200mm tube lens (Thorlabs) and planar optical microscopy lens (Olympus). Emission studies were carried out using a custom-built tilting fluorescence microscope with a planar objective (10x Olympus), a tube lens (200mm, Thorlabs), camera (Allied vision) and a fluorescence cube (Thorlabs) containing a UV filter (Thorlabs MD416) and MDF-BFP dichroic filter. The setup is designed for tracking perylene emission between 425-475 nm, via filtering out the excitation light using the fluorescence cube.

1.5 Contact angle analysis

In order to analyze contact angles of gravity-aligned Janus droplets, sideview micrographs were recorded. From the obtained micrographs, the triple phase contact line was used to determine droplet's contact angle, as a quantitative description of droplet morphology. To this end, the distance between the droplet radius and the radius of the internal curvature was recorded and used to determine the contact angle, as well as the surface area by employing Neumann construction along with the law of cosines.[4] Additionally, a correction factor $R_{real} = n_{medium}/n_{outer}R_{image}$ was implemented due to the refractive index contrast of the individual emulsion phases. All the contact angles were determined using imaging software Fiji.

1.6 Ratiometric angle-dependent emission detection setup

Prepared droplets were placed on the sample holder (Thermo-Fisher Scientific Invitrogen Attofluor Cell Chamber) by first deposing 1 mL of surfactant followed by a deposition of a droplet monolayer (20 µL) in the center of the sample holder. The sample holder was then placed on a RPS-SMA sample stage. Optical fibers were fixed on top of the sample holder (13mm above the stage) using adjustable fiber optic probe stand (Thorlabs, RPA-SMA). One of the fibers (Thorlabs, 400µm) was placed at 45° to the sample holder and used to record sideway emission. A second bifurcated fiber (Thorlabs, 400µm) was placed vertically (at 0°) above the sample holder and attached to both the spectrometer and the light source (395nm LED light from Thorlabs). Light output was recorded using a spectrometer from Avantes (model*: StarLine AvaSpec-ULS2048CL-EVO-RS*). Morphology-dependent changes in perylene emission were recorded using both fibers 0° and 45° and recording changes in the 472nm emission peak of perylene. Subsequently, the ratio of emission intensity recorded by the fiber placed at 0° over emission intensity from the fiber placed at 45° was calculated and then normalized, where value of 1 corresponded to fully encapsulated H/F/W morphology obtained by preparing droplets in a pure Zonyl FS-300 solution.

1.7 Bacteria culture:

***Salmonella enterica*:**

S. enterica DSM 554 was purchased from DSMZ. The culture was prepared in tryptic soy broth (TBS), where 5ml of TSB was inoculated with *S. enterica* from a frozen glycerol stock and incubated at 37^oC overnight. Late-logarithmic phase was obtained, with an OD_{600} value reaching ca. 0.8. The sample was diluted to an OD_{600} of 0.4 and incubated for ca. 3 hours until the desired OD of 0.65 was reached, which corresponds to app. 10^9 CFU/ml (calculated from serial agar plate dilution assays). Subsequently, serial dilutions in TBS were carried out, giving concentrations between 1- 10^8CFU/ml. From that, 100 µL of each diluted bacteria solution was transferred to 900 µL of surfactant **1** and zonyl-FS300 solution in PBS containing 300 µL of 1wt% of surfactant **1** – and 500 µL of 0.2wt% Zonyl and 100 µL of PBS. Samples were then placed in the incubator at 37^oC. Subsequently, samples were placed on a heating stage along with separate vial containing 1:1 volume mixture of DEB with 2.5mM perylene and HFE7500. Vials were heated above the Tc of the oil mixture and 100 µL of oil mix was transferred into

surfactant samples. Samples were then vortexed at 2100rpm for 10s ale left to cool down to room temperature. Changes in droplet morphology were compared with blank samples, where 100 µL of bacterial sample was replaced with 100 µL of PBS. Each sample was measured after different time points: 1h, 2h, 4h and 6h. The time required for surfactant cleavage by commercial enzyme sensing cannot be directly translated into time needed for bacteria to cleave the surfactant, because bacterial enzymes are continuously produced - meaning that the amounts of enzymes will vary between samples. Our method relies on continuous enzyme production by live bacteria. Thus, the longer incubation time, the more bacteria in the sample, the more enzymes are produced leading to larger extent of surfactant cleavage. Morphology changes were then recorded using a portable phone microscope and optical setup. All data points were measured in triplicates.

***Listeria monocytogenes*:**

L. monocytogenes DSM 20600 was purchased with DSMZ. The cultures were prepared in brain heart infusion (BHI) broth from a frozen glycerol stock. Culture was grown at 37°C for 20 hours until desired OD_{600} of 0.65 was reached, corresponding to $8*10^8$ CFU/ml.[5] Subsequently serial dilutions ranging from 10^2 CFU/mL to 10^8 CFU/mL were prepared by placing broth containing bacteria to appropriate amounts BHI broth. From that, 100 µL of each diluted bacteria solution was transferred to 900 µL of surfactant **2** and zonyl-FS300 solution in PBS containing 300 µL of 1wt% of surfactant **2** – and 500 µL of 0.2wt% Zonyl and 100 µL of PBS. Samples were then placed in the incubator at 37°C. Subsequently, samples were placed on a heating stage along with separate vial containing 1:1 volume mixture of DEB with 2.5mM perylene and HFE7500. Vials were heated above the Tc of the oil mixture and 100 µL of oil mix was transferred into surfactant samples. Samples were then vortexed at 2100rpm for 10s ale left to cool down to room temperature. Changes in droplet morphology were compared with blank samples, where 100 µL of bacterial sample was replaced with 100 µL of PBS. Each sample was measured after different time points: 1h, 2h, 4h and 6h. The time required for surfactant cleavage by commercial enzyme sensing cannot be directly translated into time needed for bacteria to cleave the surfactant, because bacterial enzymes are continuously produced - meaning that the amounts of enzymes will vary between samples. Our method relies on continuous enzyme production by live bacteria. Thus, the longer incubation time, the more bacteria in the sample, the more enzymes are produced leading to larger extent of surfactant

cleavage. Morphology changes were then recorded using a portable phone microscope and optical setup. All data points were measured in triplicates.

Escherichia coli

E.coli DH5α was purchased from New England Biolabs. The culture was grown from a frozen glycerol stock in lysogeny broth (LB). The culture was incubated at 37°C overnight. The next day the culture was diluted to an OD_{600} of 0.6 and allowed to grow to an OD_{600} of 1.0, which corresponds to app. 10^9 CFU/ml.[6] Subsequently serial dilutions ranging from 10^2 CFU/mL to 10^8 CFU/mL were prepared by placing broth containing bacteria to appropriate amounts LB broth. From that, 100 µL of each diluted bacteria solution was transferred to 900 µL of surfactant **3** and zonyl-FS300 solution in PBS containing 400 µL of 1wt% Surfactant **3** – and 500 µL of 0.2wt% Zonyl. Samples were then placed in the incubator at 37°C. Subsequently, samples were placed on a heating stage along with separate vial containing 1:1 volume mixture of DEB with 2.5mM perylene and HFE7500. Vials were heated above the Tc of the oil mixture and 100 µL of oil mix was transferred into surfactant samples. Samples were then vortexed at 2100rpm for 10s and left to cool down to room temperature. Changes in droplet morphology were compared with blank samples, where 100 µL of bacterial sample was replaced with 100 µL of PBS. Each sample was measured after different time points: 1h, 2h, 4h and 6h. The time required for surfactant cleavage by commercial enzyme sensing cannot be directly translated into time needed for bacteria to cleave the surfactant, because bacterial enzymes are continuously produced - meaning that the amounts of enzymes will vary between samples. Our method relies on continuous enzyme production by live bacteria. Thus, the longer incubation time, the more bacteria in the sample, the more enzymes are produced leading to larger extent of surfactant cleavage. Morphology changes were then recorded using a portable phone microscope and optical setup. All data points were measured in triplicates.

2. ^{1}H and ^{13}C NMR spectra of synthesized surfactants 1-3

2.1 ^{1}H and ^{13}C NMR spectra of surfactants 1-3

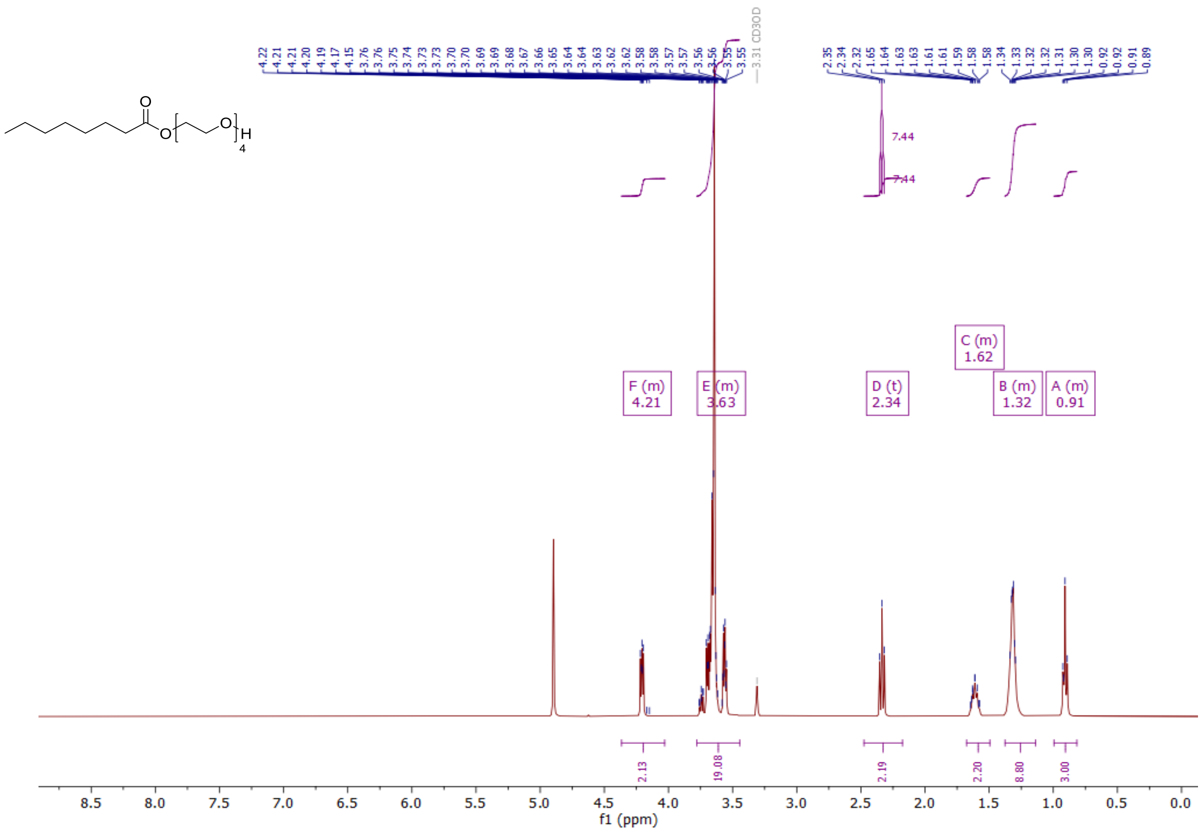

Fig. S1 ^{1}H NMR of synthesized surfactant **1** (400 MHz; MeOD-d4)

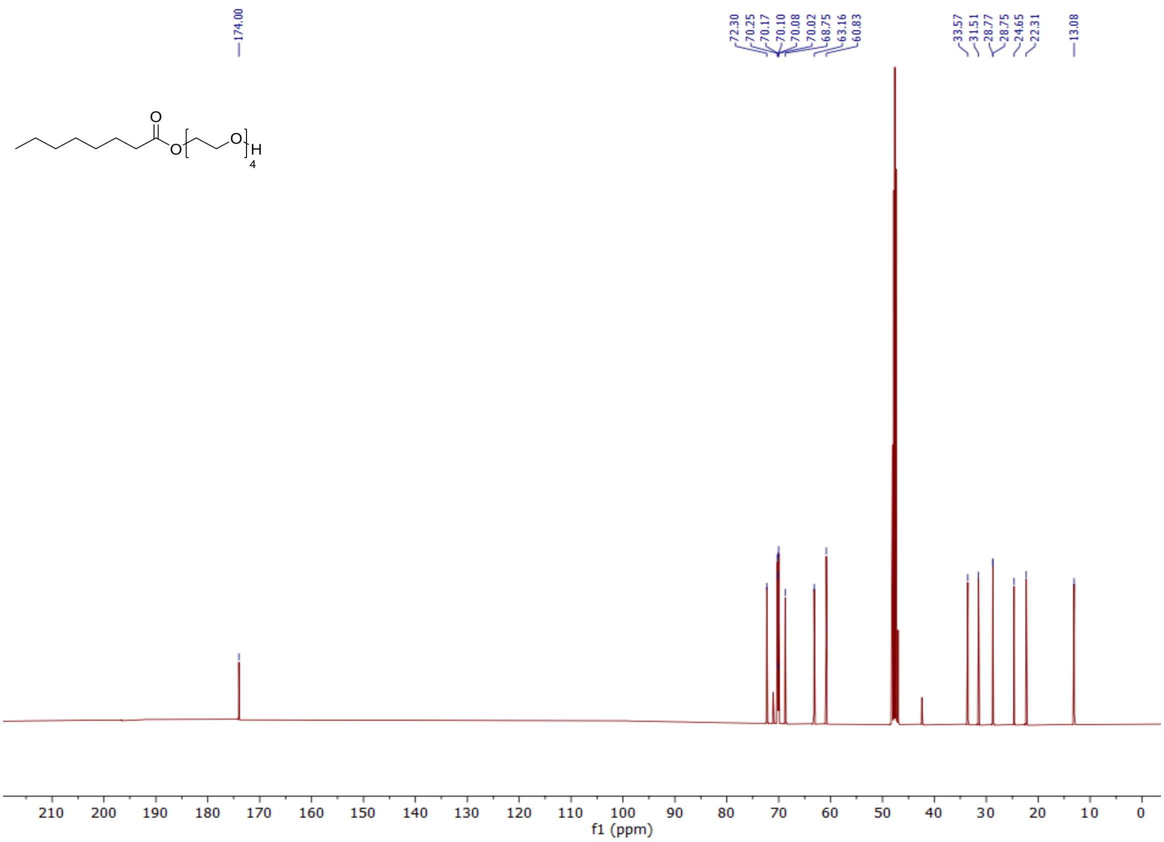

Fig. S2 ^{13}C NMR of synthesized surfactant **1** (400 MHz; MeOD-d_4)

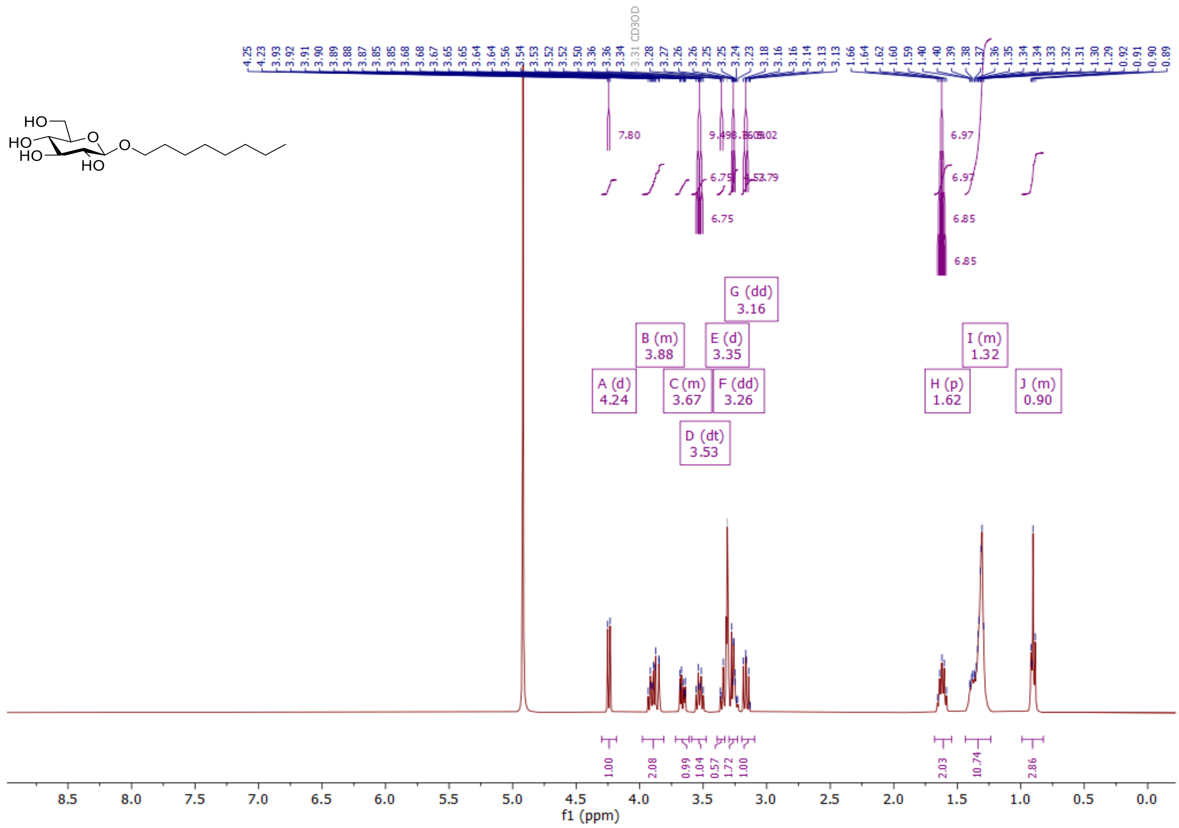

Fig. S3 ^{1}H NMR of synthesized surfactant **2** (400 MHz; MeOD-d_4)

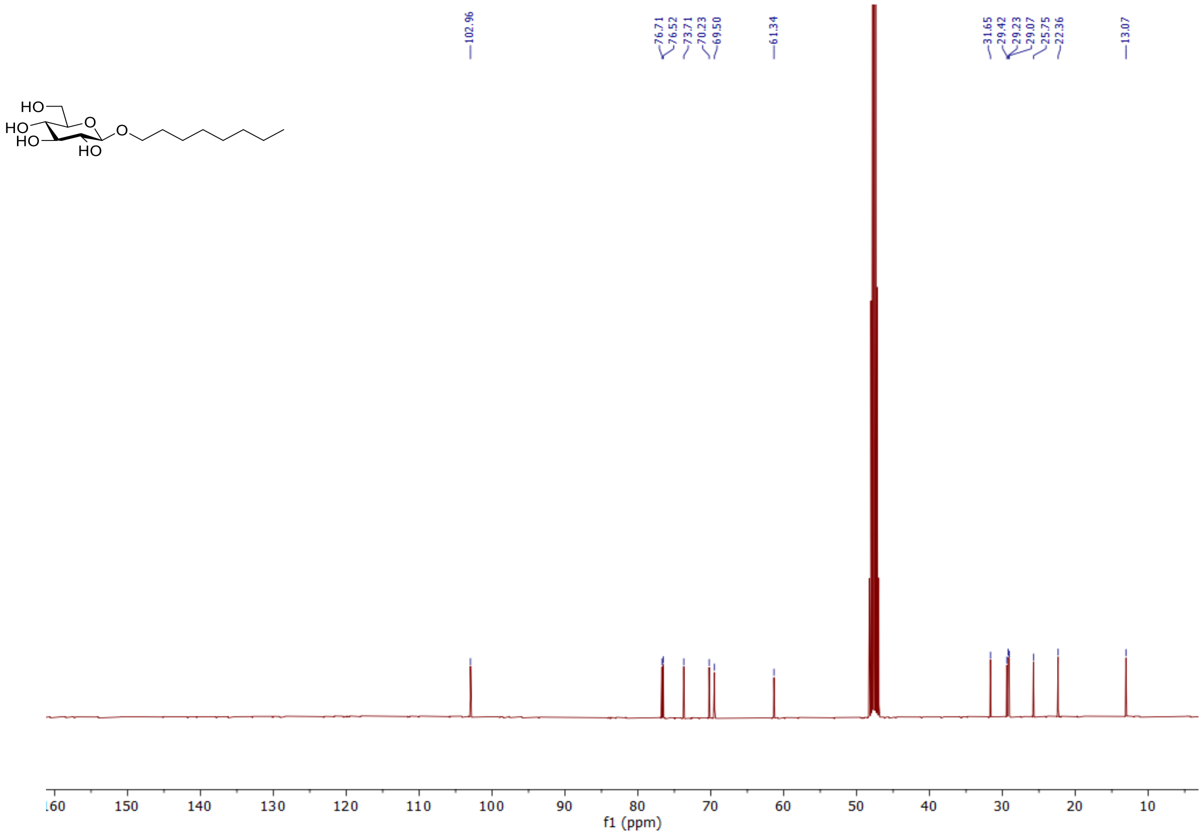

Fig. S4 ^{13}C NMR of synthesized surfactant **2** (400 MHz; MeOD-d$_4$)

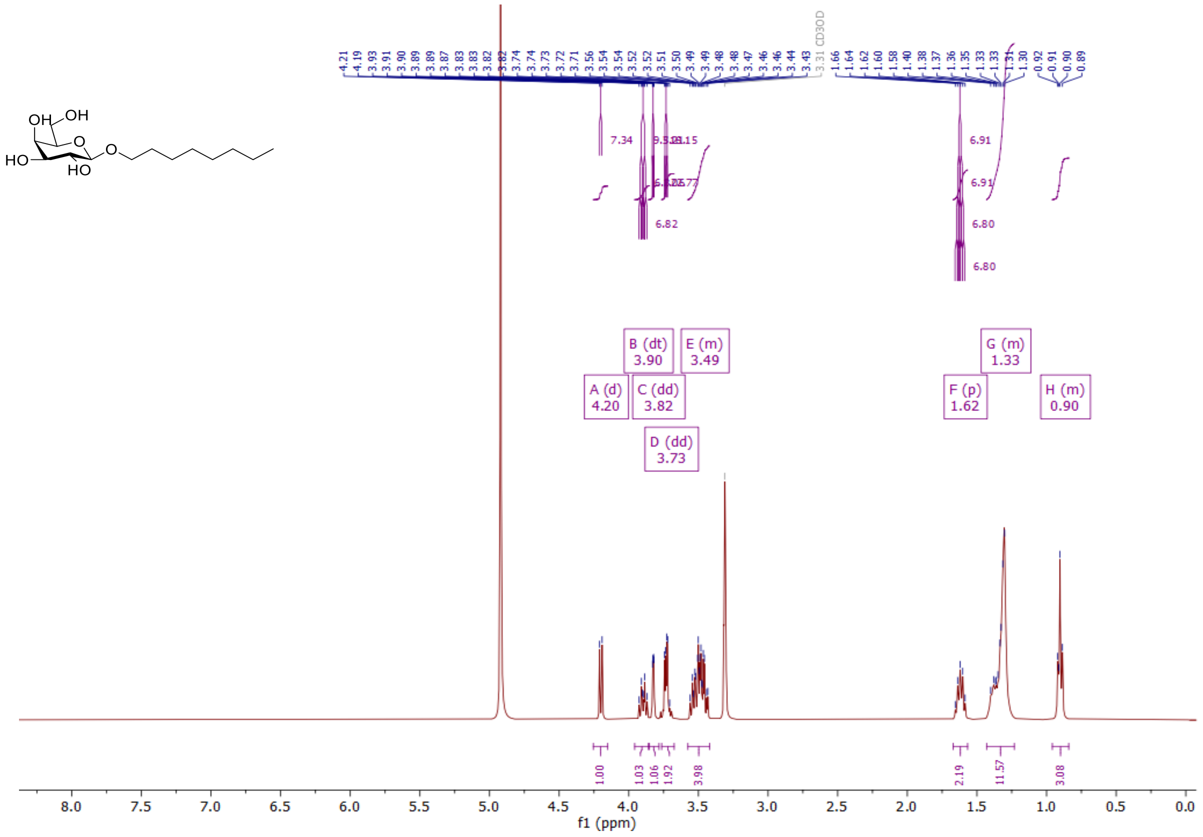

Fig. S5 ^{1}H NMR of synthesized surfactant **3** (400 MHz; MeOD-d$_4$)

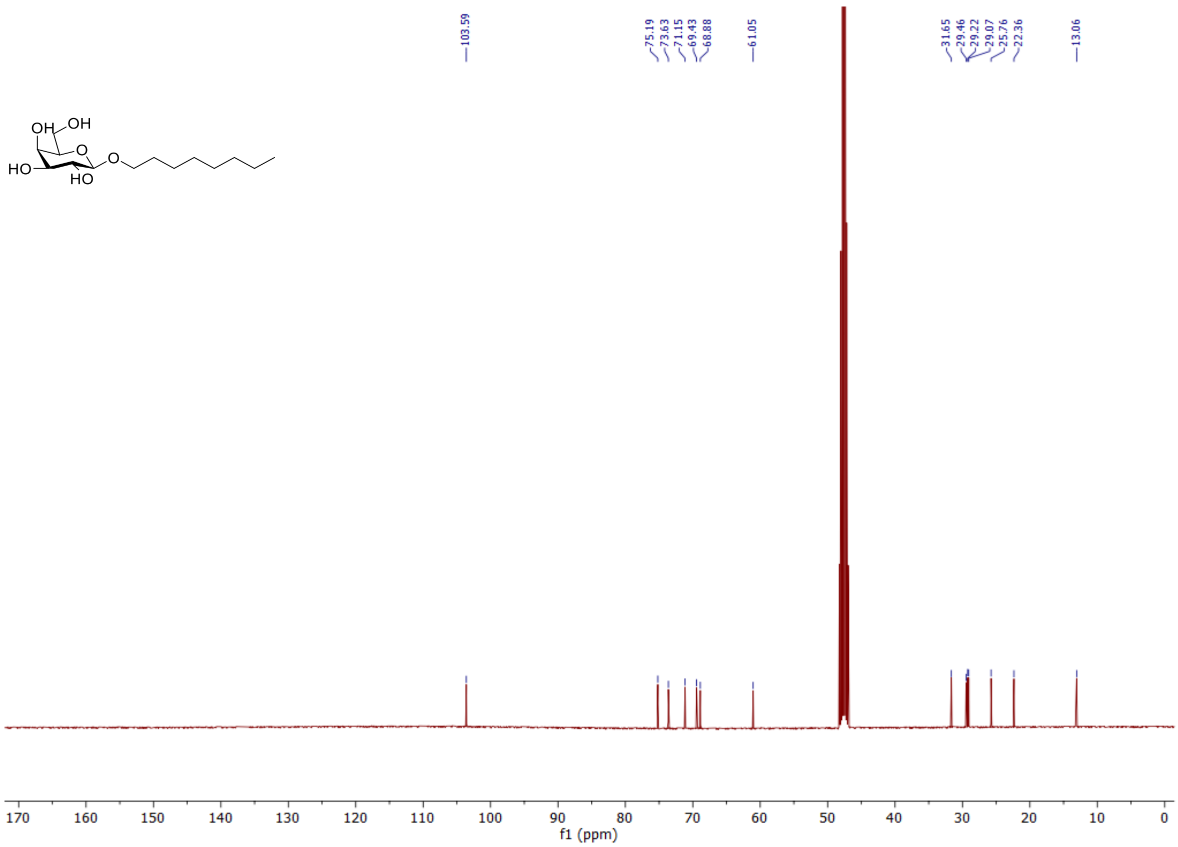

Fig. S6 ^{13}C NMR of synthesized surfactant **3** (400 MHz; MeOD-d$_4$)

2.2 ^{1}H NMR cleavage studies – time dependency:

^{1}H NMR investigation of enzymatic cleavage was carried out via monitoring variations in the respective NMR spectra of the surfactants upon incubation with the respective enzymes. Solution of surfactant **1** with PLE, **2** with β-glucosidase and **3** with β-galactosidase were prepared in D_2O and checked after different time points. Between measurements, samples were placed on a shaker to ensure maximum exposure to the enzyme. Concentration of each surfactant was kept constant as 0.1wt% and enzyme concentration was kept constant as 1 U/mL. Shifts highlighted on the ^{1}H NMR spectra listed below, indicate the extent of the cleavage. Obtained spectra were compared with uncleaved surfactant spectrum and spectrum of cleaved hydrophilic part of a surfactant, i.e. tetraethylene glycol for **1**, glucose for **2** and galactose for **3**. For cleavage of **1** by PLE, already after 30 minutes more than 50% of the surfactant is consumed. After 2 hours cleavage is finished. Cleavage of **2** by β-glucosidase reached 50% after 30 minutes and was completed after 2 hours. Cleavage of **3** was completed after 4 hours.

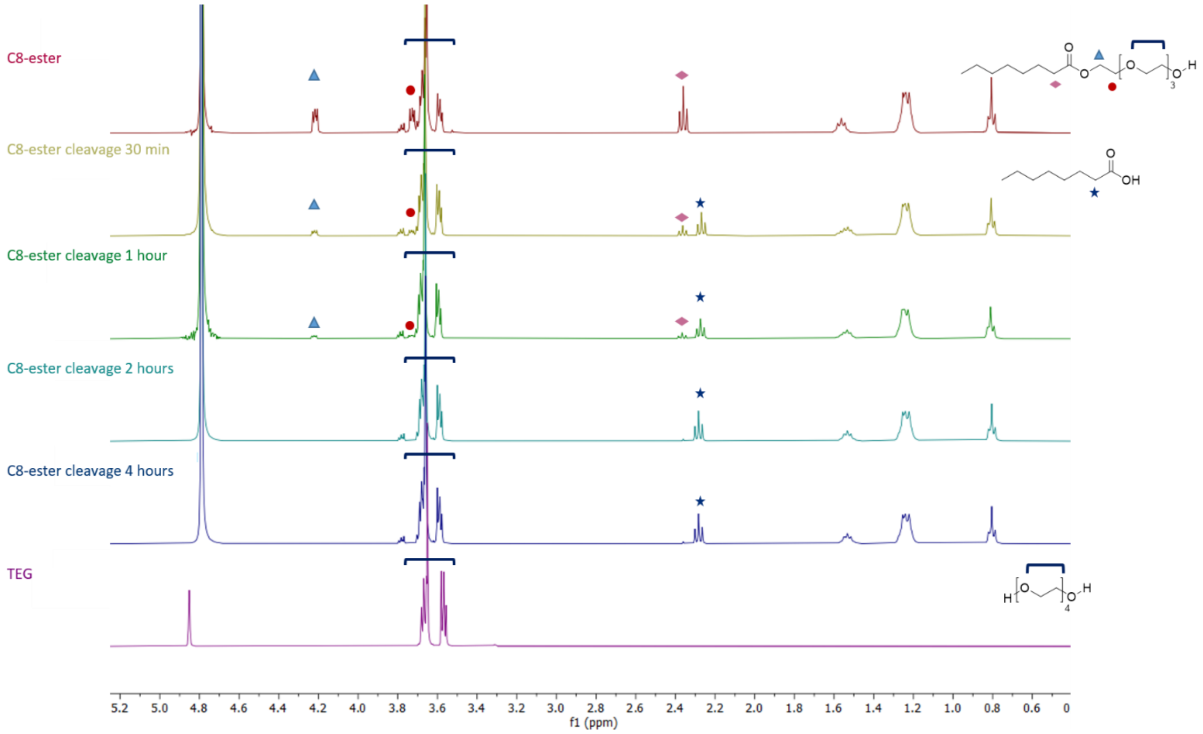

Fig. S7 Time-dependence of cleavage of surfactant **1** with PLE (D_2O)

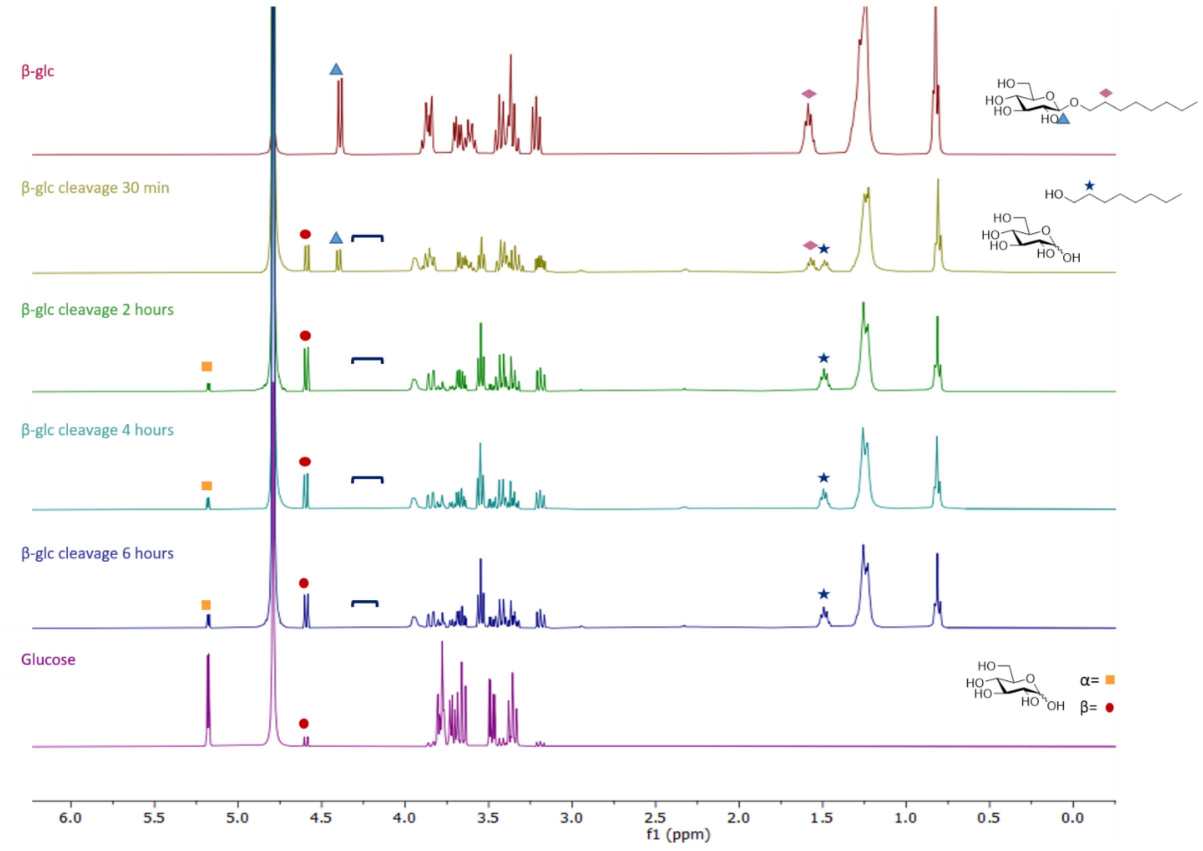

Fig. S8 Time-dependence of cleavage of surfactant **2** with β-glucosidase (D_2O)

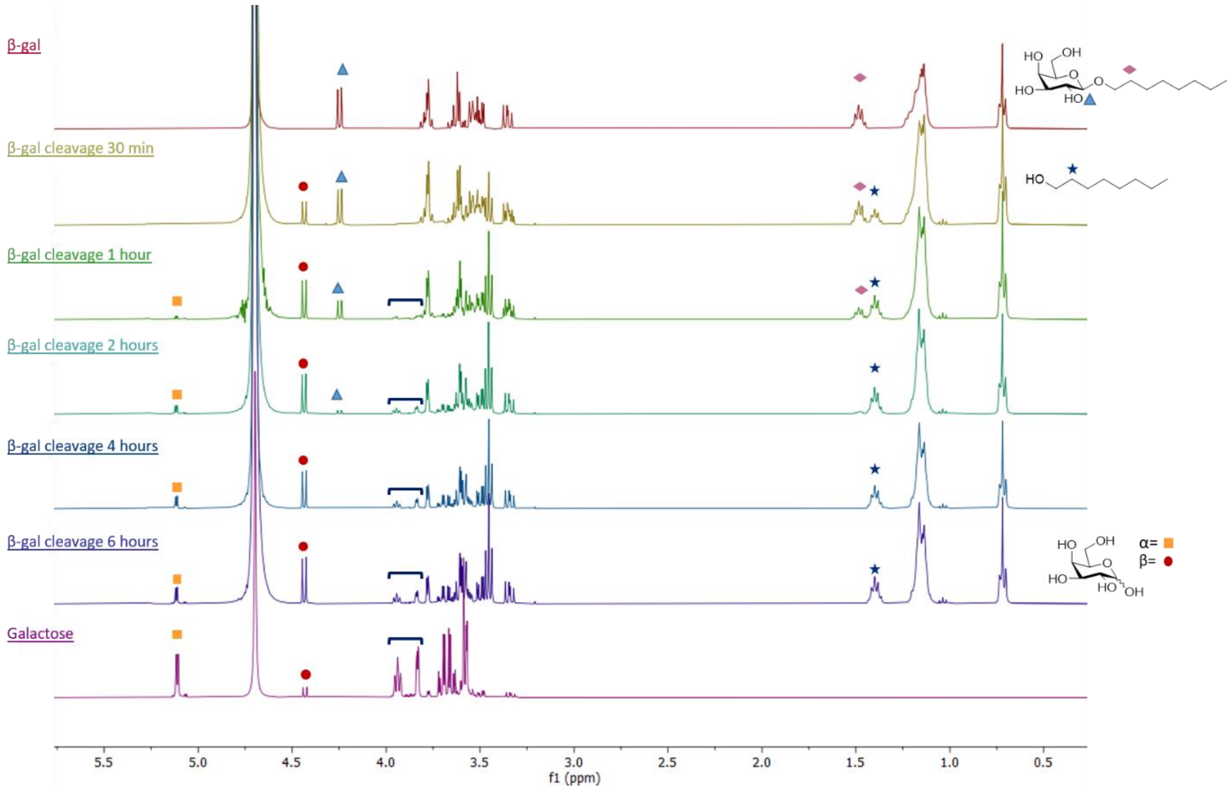

Fig. S9 Time-dependence of cleavage of surfactant **3** with β-galactosidase (D_2O)

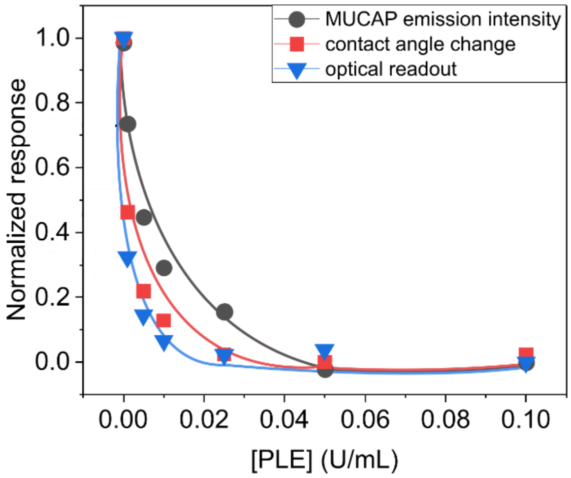

Fig. S10 Enzyme (PLE) concentration-dependent response of the cleavage of surfactant **1** determined via Janus droplet morphological changes (red) and via dual angle-dependent ratiometric recording of the droplet emission signature (blue), both compared with the commercial Mucap® test that is based on enzymatic hydrolysis of a chemiluminescent 4-methylumbelliferyl-probe. Both tests are based on molecular cleavage by the target enzymes. However the transduction mechanism is different. Chmiluminescent probes require concentrations of fluorecent probes above the fluorescence detection threshold. In contrast, modular Janus droplet transducers serve to visualize marginal variations in the balance of interfacial tensions induced by surfactant cleavage. Considering the very small interfacial area of one hemisphere of Janus droplets and that variations on the order in 0.5 mN m^{-1} suffice to induce morphological changes on the order of 20° (that suffice for a full optical response),[7] the theoretical limits of detection of the latter can become very low, particularly when operated below the cmc of the target surfactants.

2.3 ^{1}H NMR cleavage studies – enzymatic cross tests:

Surfactants kept at concentration of 0.1wt% and enzymes at 1U/ml. Progress of the cross cleavage was measured after different time points.

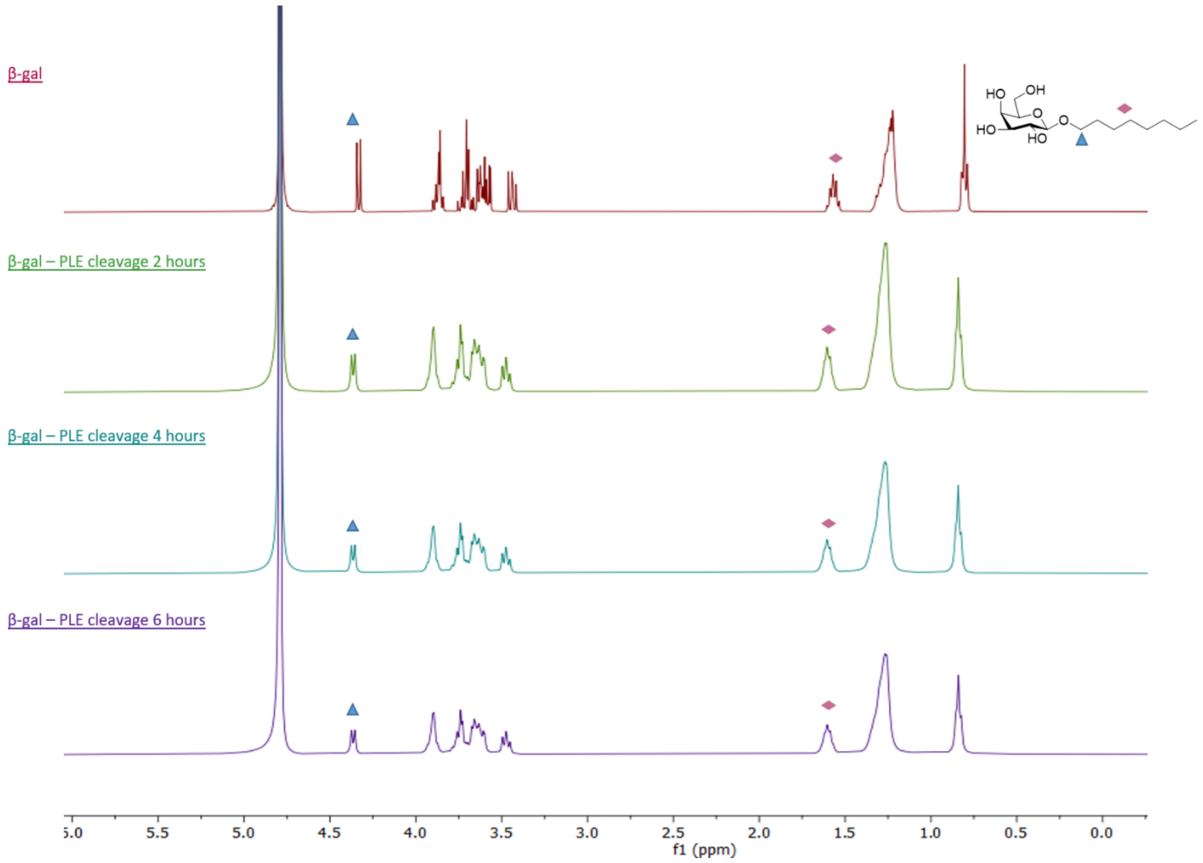

Fig. S11 PLE vs surfactant **3**

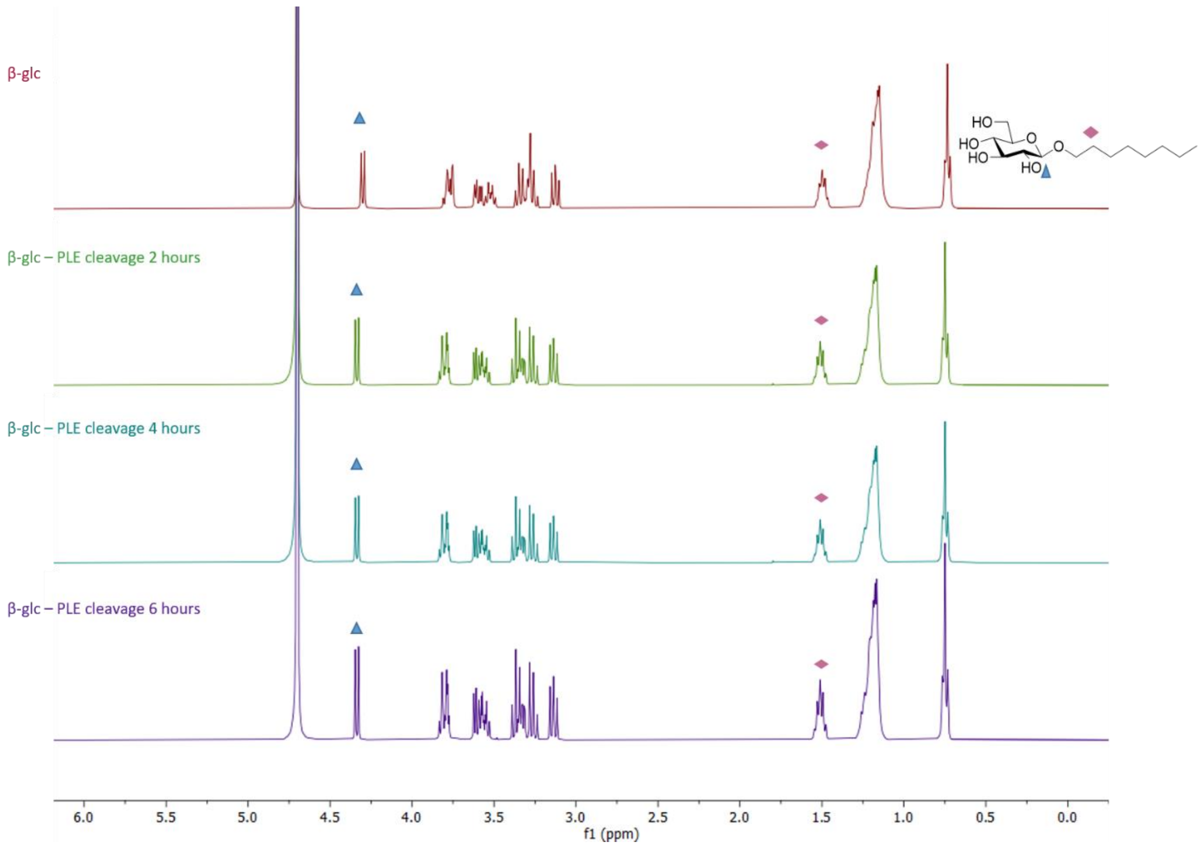

Fig. S12 PLE vs surfactant **2**

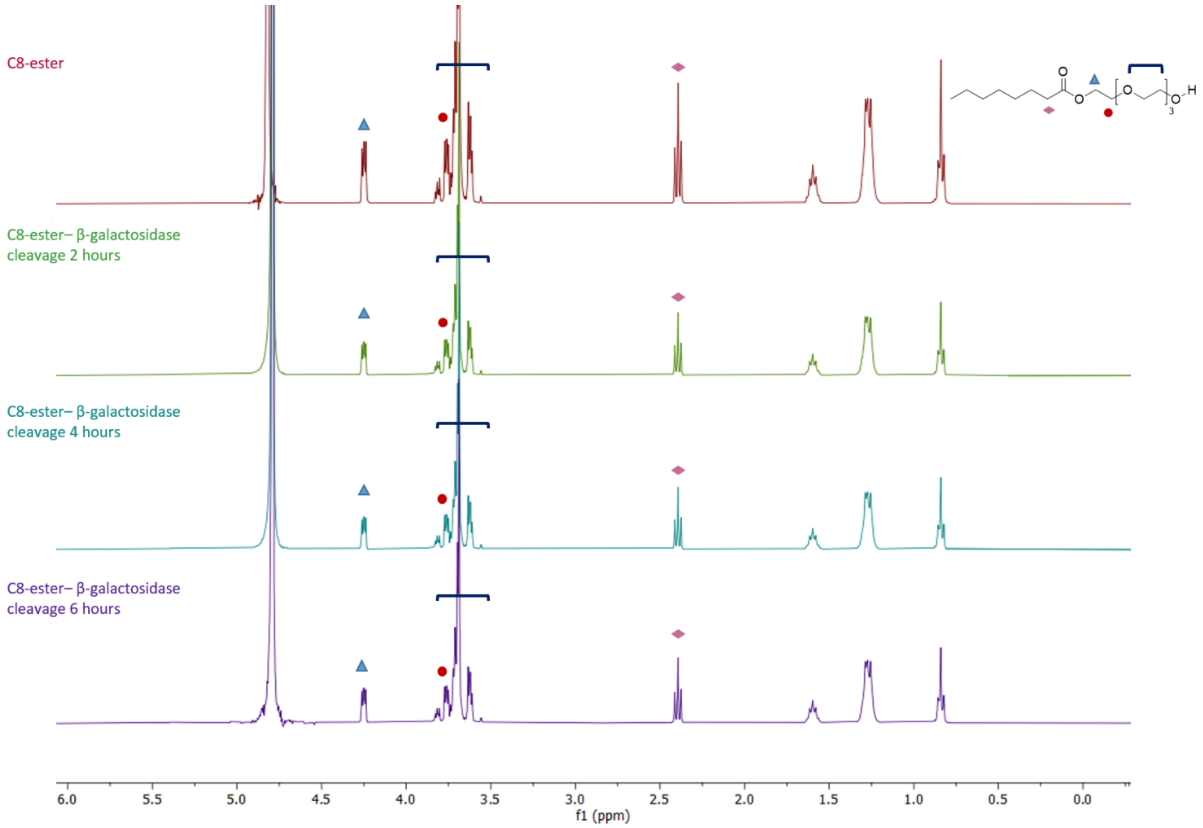

Fig. S13 β-galactosidase vs surfactant **1**

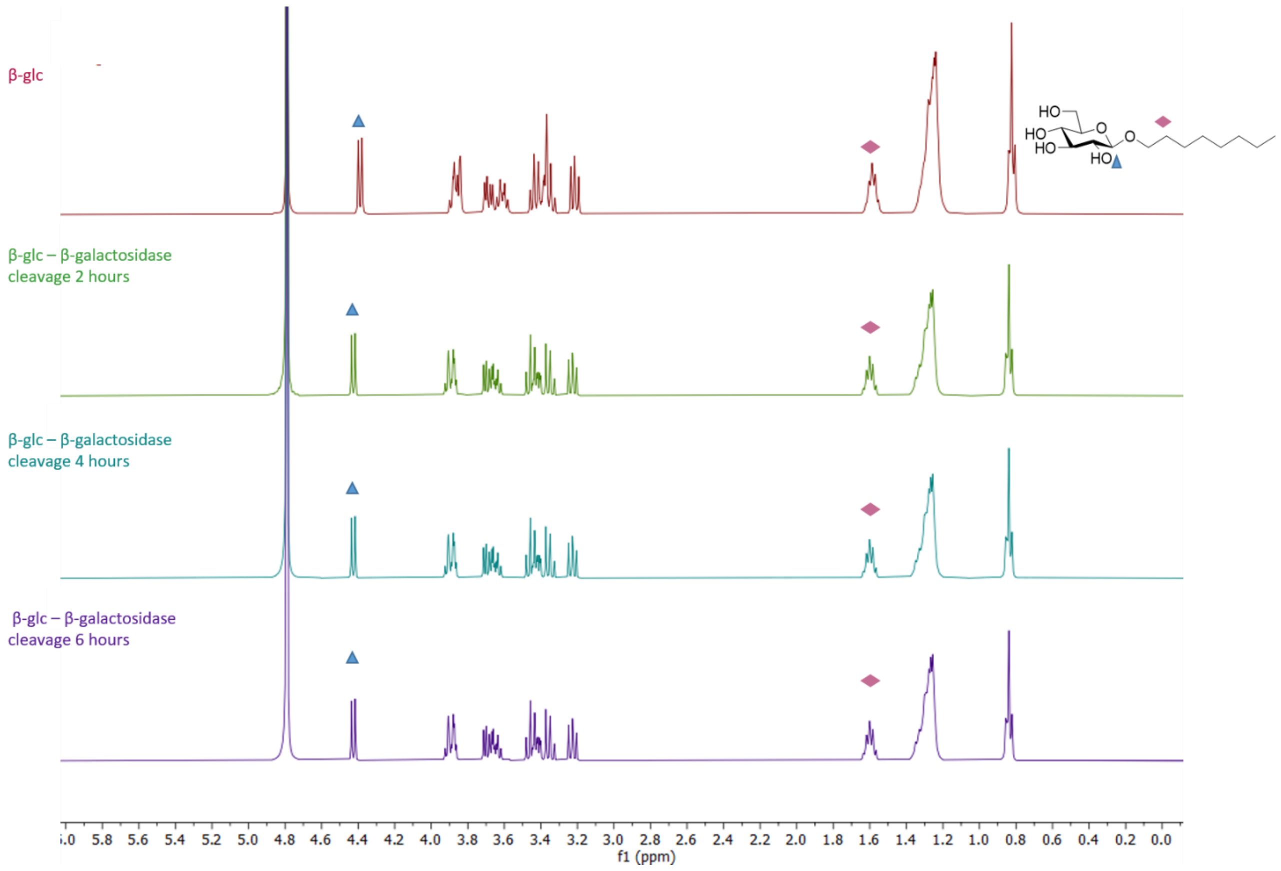

Fig. S14 β-galactosidase vs surfactant **2**

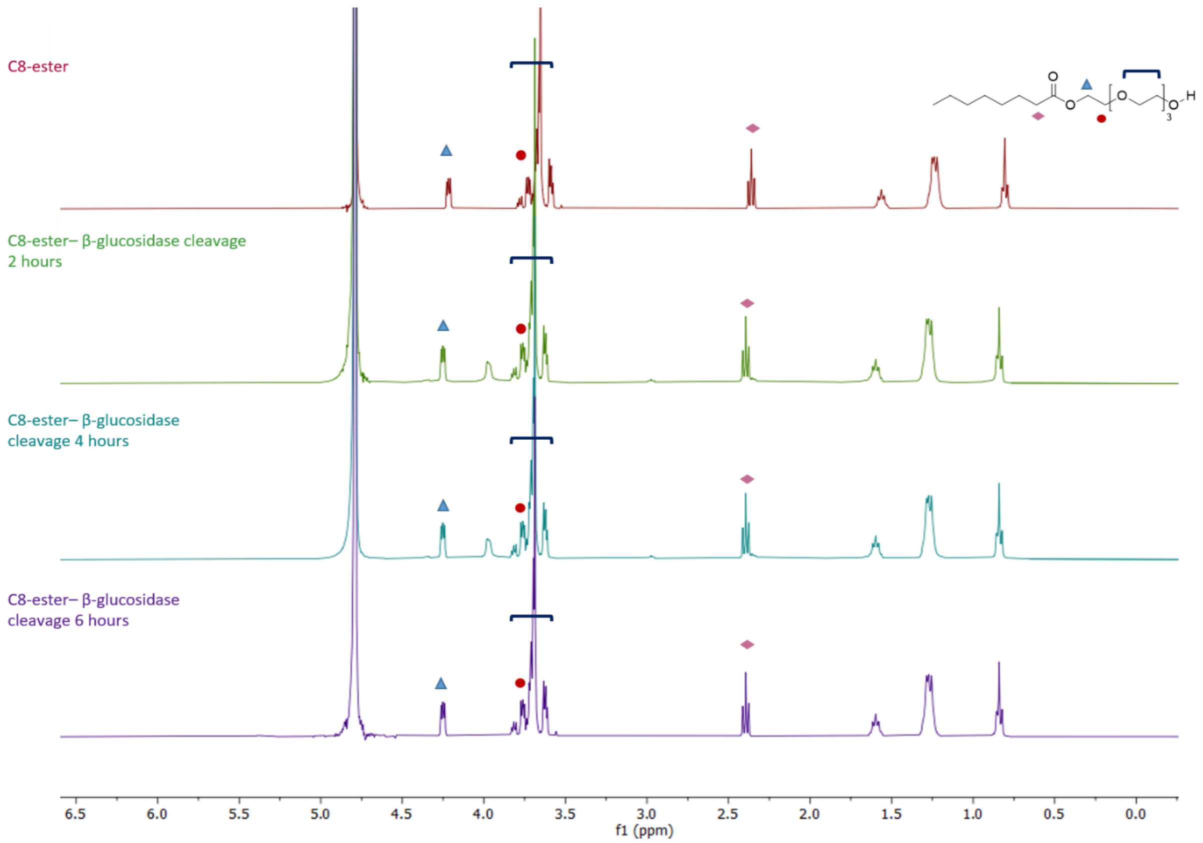

Fig. S15 β-glucosidase vs surfactant **1**

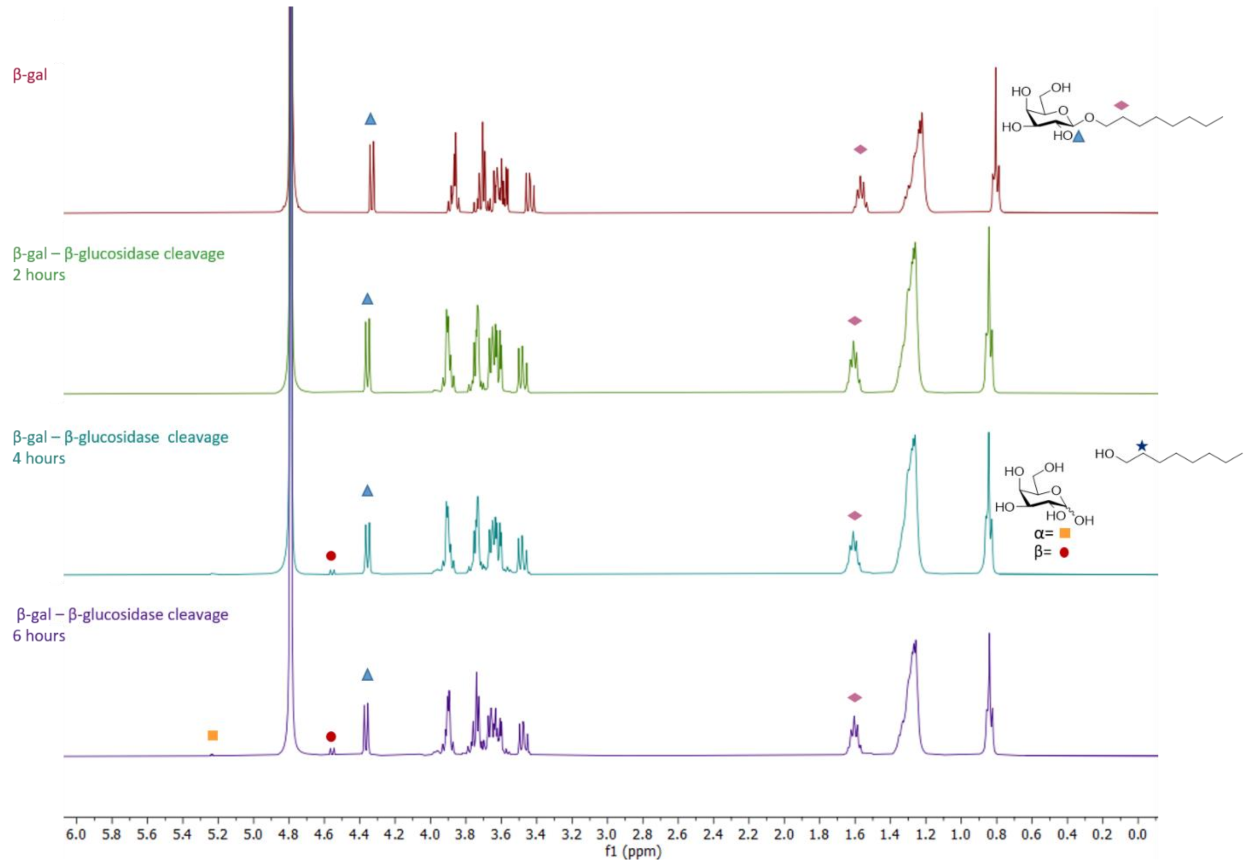

Fig. S16 β-glucosidase vs surfactant **3**

3. Detection of enzymatic activity using Janus double emulsions

For all cleavage studies, enzymes were added and incubated with an appropriate surfactant mixture prior to emulsification to avoid any additional interactions between droplet oils and enzymes. The same approach was employed throughout bacteria detection studies.

Porcine liver esterase (PLE): experiments were conducted at room temperature in PBS at pH 7.4. All solutions, including surfactant stocks and enzyme stocks were prepared using PBS. Experiments have been carried out with a constant surfactant concentration (300 µL of 1wt% of surfactant **1** – and 500 µL of 0.2wt% Zonyl and 100 µL of PBS) against varying PLE concentrations (100 µL) after 2 hours or different reaction times using 0.025 U/mL of final PLE concentration. After enzyme addition, samples were placed on a shaker to ensure correct stirring and even enzyme distribution throughout the sample. Subsequently, samples were placed on a heating stage along with separate vial containing 1:1 volume mixture of DEB with 2.5mM perylene and HFE7500. Vials were heated above the Tc of the oil mixture and 100 µL of oil mix was transferred into surfactant samples. Samples were then vortexed at 2100rpm for 10s ale left to cool down to room temperature. Changes in droplet morphology were compared with blank samples, where 100 µL of enzyme solution was replaced with 100 µL of PBS. Morphology changes were then recorded using a sideview microscope and optical setup. All data points were measured in triplicates.

β-glucosidase: experiments were conducted at room temperature in PBS at pH 7.4. All solutions, including surfactant stocks and enzyme stocks were prepared using PBS. Experiments have been carried out with a constant surfactant concentration (300 µL of 1wt% of surfactant **2** – and 500 µL of 0.2wt% Zonyl and 100 µL of PBS) against varying β-glucosidase concentrations after 2 hours or different reaction times using 1 U/mL of final β-glucosidase concentration. After enzyme addition, samples were placed on a shaker to ensure correct stirring and even enzyme distribution throughout the sample. Subsequently, samples were placed on a heating stage along with separate vial containing 1:1 volume mixture of DEB with 2.5mM perylene and HFE7500. Vials were heated above the Tc of the oil mixture and

100 µL of oil mix was transferred into surfactant samples. Samples were then vortexed at 2100rpm for 10s ale left to cool down to room temperature. Changes in droplet morphology were compared with blank samples, where 100 µL of enzyme solution was replaced with 100 µL of PBS. Morphology changes were then recorded using a sideview microscope and optical setup. All data points were measured in triplicates.

β-galactosidase: experiments were conducted at room temperature in PBS at pH 7.4. All solutions, including surfactant stocks and enzyme stocks were prepared using PBS. Experiments have been carried out with a constant surfactant concentration (400 µL of 1wt% Surfactant **3** – and 500 µL of 0.2wt% Zonyl) against varying β-galactosidase concentrations after 2 hours or different reaction times using 2.5 U/mL of final β-galactosidase concentration. After enzyme addition, samples were placed on a shaker to ensure correct stirring and even enzyme distribution throughout the sample. Subsequently, samples were placed on a heating stage along with separate vial containing 1:1 volume mixture of DEB with 2.5mM perylene and HFE7500. Vials were heated above the Tc of the oil mixture and 100 µL of oil mix was transferred into surfactant samples. Samples were then vortexed at 2100rpm for 10s ale left to cool down to room temperature. Changes in droplet morphology were compared with blank samples, where 100 µL of enzyme solution was replaced with 100 µL of PBS. Morphology changes were then recorded using a sideview microscope and optical setup. All data points were measured in triplicates.

3.1 Determination of the specificity of the system – enzymatic cross tests

Cross cleavage was investigated by incubating surfactants with enzymes other than their targets.

PLE - 0.025 U/mL of enzyme solution in PBS solution (100 µL) was added to 900 µL of surfactant solution containing 300 µL of 1wt% of surfactant **2** – and 500 µL of 0.2wt% Zonyl and 100 µL of PBS. The same procedure was carried out for surfactant **3**: 0.025U/mL of enzyme solution in PBS solution (100 µL) was added to 900 µL of surfactant solution containing 400 µL of 1wt% Surfactant **3** – and 500 µL of 0.2wt% Zonyl. Samples were then placed on a shaker for 2 hours. Subsequently, samples were placed on a heating stage along with separate vial containing 1:1 volume mixture of DEB with 2.5mM perylene and HFE7500. Vials were heated above the Tc of the oil mixture and 100 µL of oil mix was transferred into surfactant samples. Samples were then vortexed at 2100rpm for 10s and left to cool down to room temperature. Changes in droplet morphology were compared with blank samples, where 100 µL of enzyme solution was replaced with 100 µL of PBS. Morphology changes were then recorded using a sideview microscope and optical setup. All data points were measured in triplicates.

β-glucosidase: - 1 U/mL of of enzyme solution in PBS solution (100 µL) was added to 900 µL of surfactant solution containing 300 µL of 1wt% of surfactant **1** – and 500 µL of 0.2wt% Zonyl and 100 µL of PBS. The same procedure was carried out for surfactant **3**: 1 U/mL of enzyme solution in PBS solution (100 µL) was added to 900 µL of surfactant solution containing 400 µL of 1wt% Surfactant **3** – and 500 µL of 0.2wt% Zonyl. Samples were then placed on a shaker for 2 hours. Subsequently, samples were placed on a heating stage along with separate vial containing 1:1 volume mixture of DEB with 2.5mM perylene and HFE7500. Vials were heated above the Tc of the oil mixture and 100 µL of oil mix was transferred into surfactant samples. Samples were then vortexed at 2100rpm for 10s and left to cool down to room temperature. Changes in droplet morphology were compared with blank samples, where 100 µL of enzyme solution was replaced with 100 µL of PBS. Morphology changes were then recorded using a sideview microscope and optical setup. All data points were measured in triplicates.

β-galactosidase: - 2.5 U/mL of enzyme solution in PBS solution (100 µL) was added to 900 µL of surfactant solution 330 µL of 1wt% of surfactant **1** – and 570 µL of 0.2wt% Zonyl. The same procedure was carried out for surfactant **2**: 2.5U/mL of enzyme solution in PBS solution (100 µL) was added to 900 µL of surfactant solution containing 420 µL of 1wt% of surfactant **2** – and 480 µL of 0.2wt% Zonyl. Samples were then placed on a shaker for 2 hours. Subsequently, samples were placed on a heating stage along with separate vial containing 1:1 volume mixture of DEB with 2.5mM perylene and HFE7500. Vials were heated above the Tc of the oil mixture and 100 µL of oil mix was transferred into surfactant samples. Samples were then vortexed at 2100rpm for 10s and left to cool down to room temperature. Changes in droplet morphology were compared with blank samples, where 100 µL of enzyme solution was replaced with 100 µL of PBS. Morphology changes were then recorded using a sideview microscope and optical setup. All data points were measured in triplicates.

3.2 Enzymatic cleavage in Janus emulsions – time dependence studies

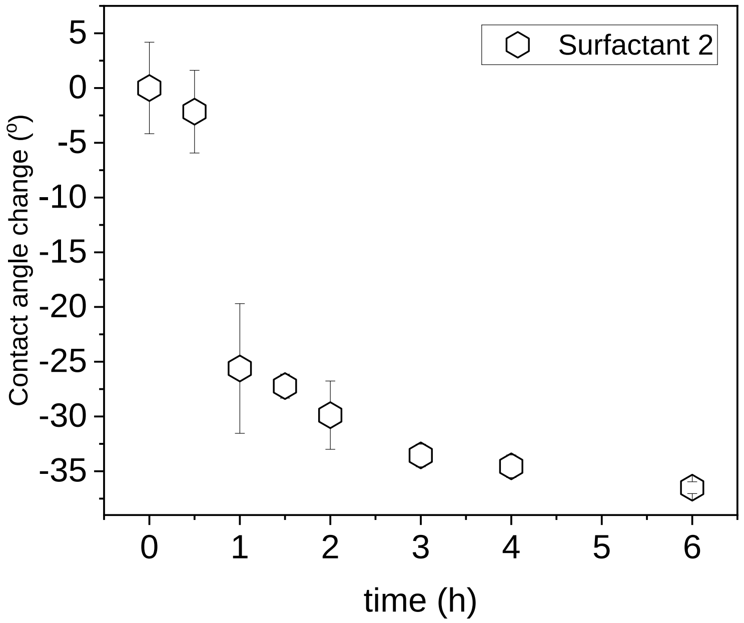

Fig. S17 Time dependence study for enzymatic cleavage of surfactant 2 (10.2 nmol/mL) with β-glucosidase (1 U/mL).

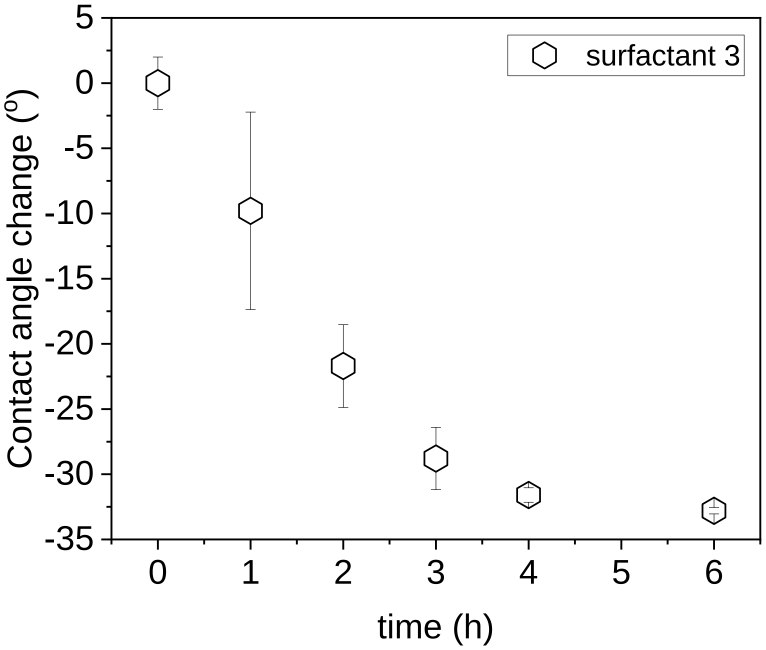

Fig. S18 Time dependence study for enzymatic cleavage of surfactant 3 (13.6 nmol/mL) with β-galactosidase (2.5 U/mL).

3.3 Enzyme kinetics:

To calculate concentration of surfactant within the emulsion system upon cleavage, droplet contact angle was plotted against HC surfactant concentration used. Plot for each surfactant shows linear behavior, therefore y=mx+b equation can be used, where x is a surfactant concentration assigned to a given droplet morphology. Those values were then applied to calculate enzyme kinetics and rate constants.

Given that enzymes exhibit first order kinetics, rate constant of the enzymatic cleavage, k, can be calculated by following equation:

$$\mathrm{Ln}[A]_t = -kt + \ln[A]_0$$

Where -k is the slope, t is the investigated reaction time, $[A]_0$ (μmol/mL) is a starting surfactant concentration and $[A]_t$ (μmol/mL) is surfactant concentration after given incubation time. Linear fit was placed and rate constants after 2 H of cleavage were compared.

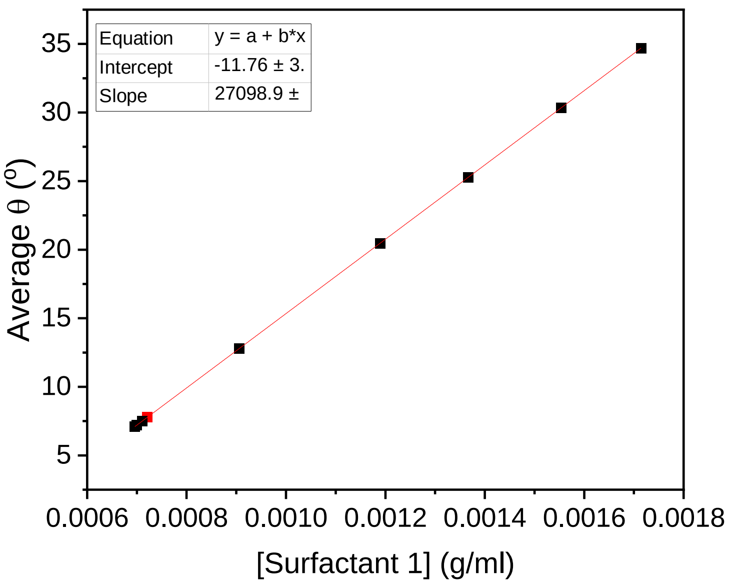

Fig. S19 Contact angle vs surfactant **1** concentration (Zonyl-FS300 concentration kept constant).

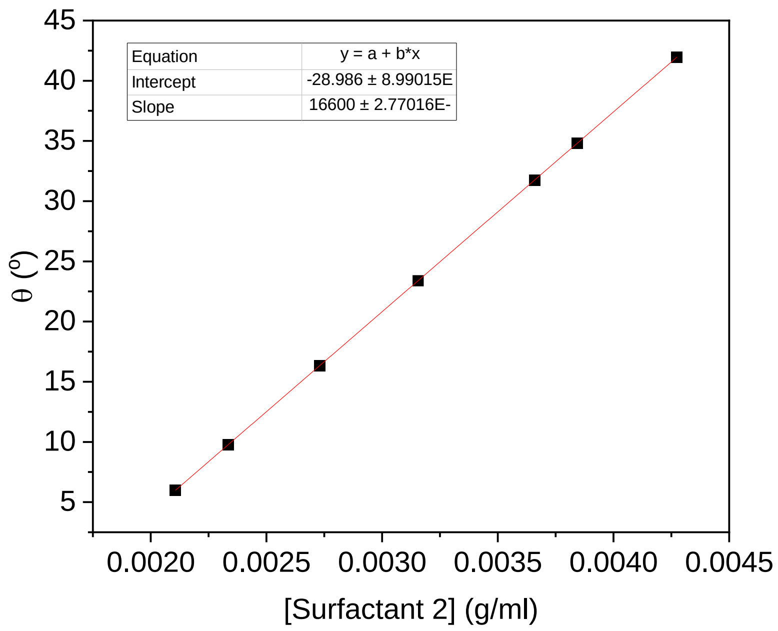

Fig. S20 Contact angle vs surfactant **2** concentration (Zonyl-FS300 concentration kept constant).

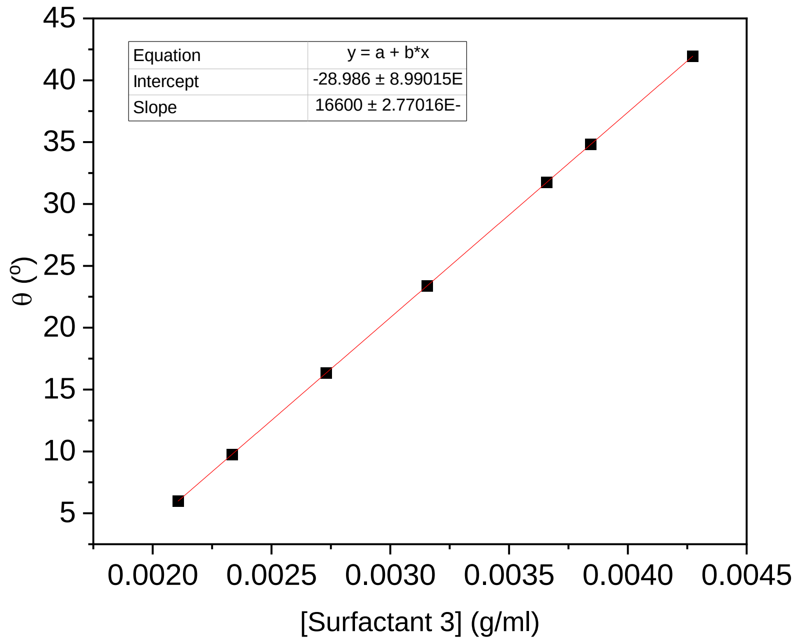

.

Fig. S21 Contact angle vs surfactant **3** concentration (Zonyl-FS300 concentration kept constant)

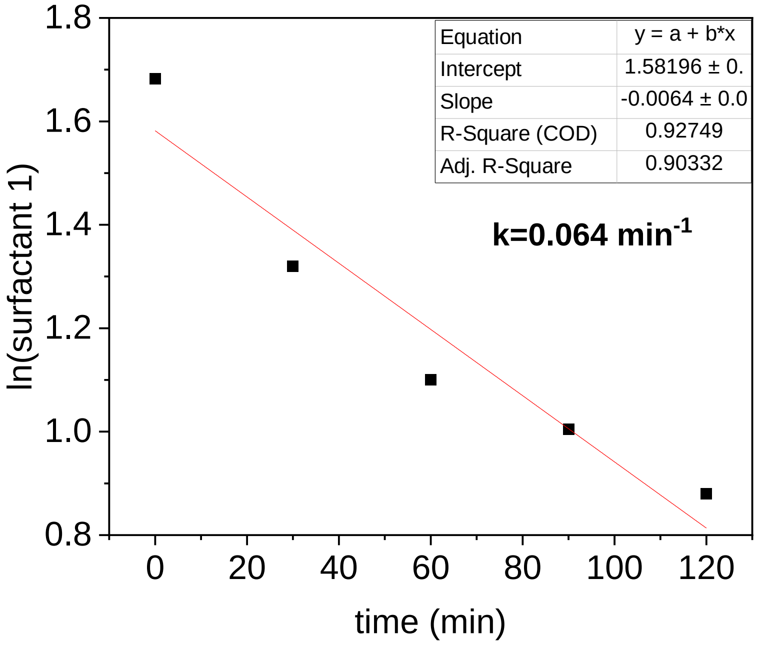

Fig. S22 PLE vs surfactant **1** – enzyme kinetics. Rate constant after 2 H of cleavage.

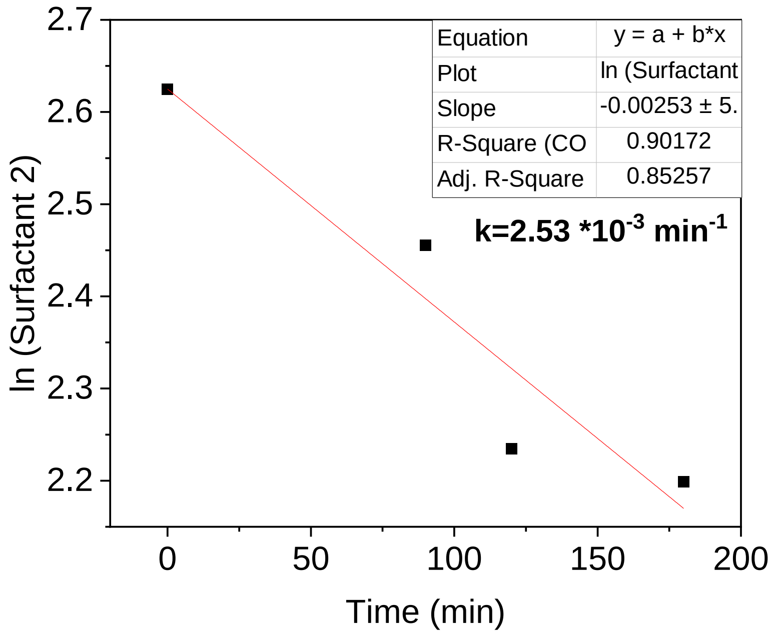

Fig. S23 β-glucosidase vs surfactant **2** – enzyme kinetics. Rate constant after 2 H of cleavage.

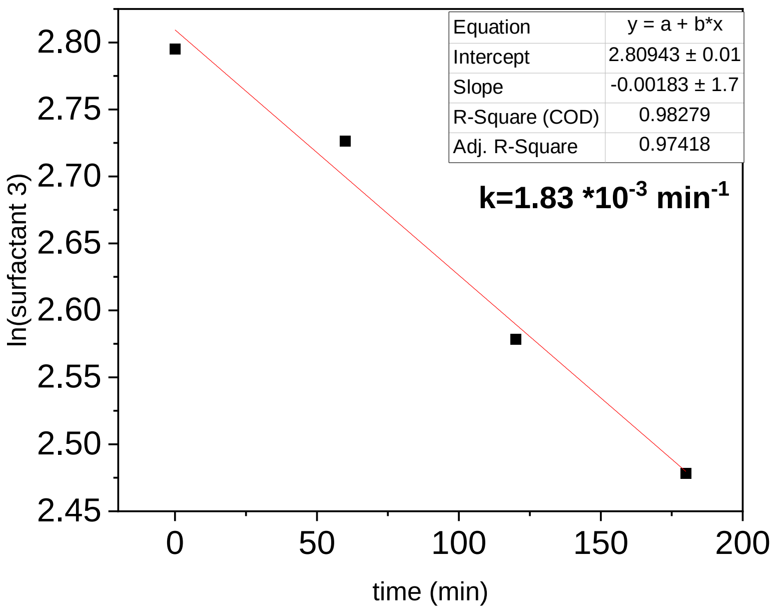

Fig. S24 β-galactosidase vs surfactant **3** – enzyme kinetics. Rate constant after 2 H of cleavage.

3.4 Enzymatic cleavage in Janus emulsions - concentration dependence studies:

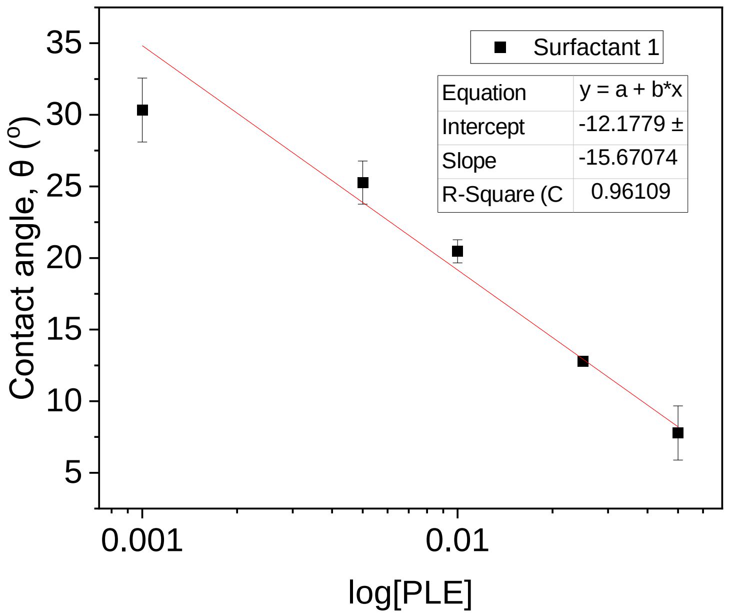

Fig. S25 Concentration dependence study showing a linear behavior between the decrease in the contact angle of droplets stabilized with surfactant **1** and PLE concentration.

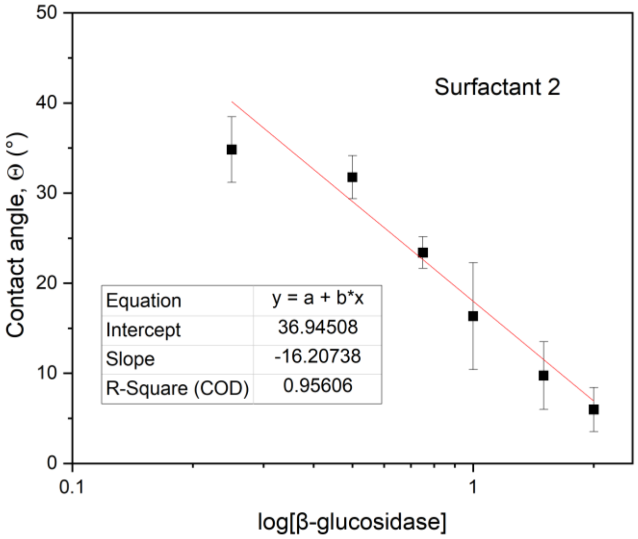

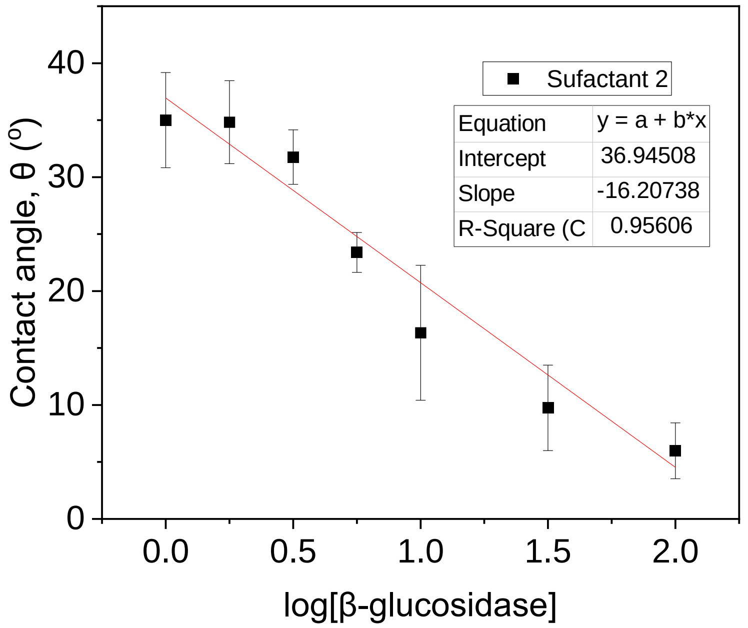

Fig. S26 Concentration dependence study showing a linear behavior between the decrease in the contact angle of droplets stabilized with surfactant **2** and β-glucosidase concentration.

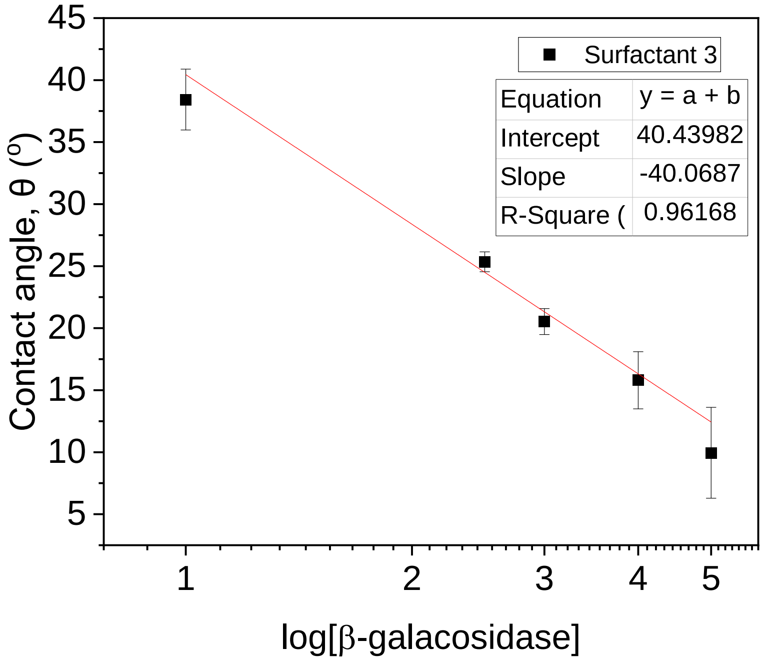

Fig. S27 Concentration dependence study showing a linear behavior between the decrease in the contact angle of droplets stabilized with surfactant **3** and β-galactosidase concentration.

4. Bacteria sensing:

4.1 General procedure for bacteria sensing with Janus emulsions:

For all bacteria studies surfactant solutions were still prepared in PBS. Surfactant concentrations used in bacteria sensing studies were the same as concentrations used for detection of commercial enzymes. To the prepared surfactant solution 100 µL of bacteria solution in a growth medium was added and the sample was placed in the incubator with shaking at 37°C. Throughout bacteria detection studies, bacterial solutions were added and incubated with an appropriate surfactant mixture prior to emulsification to avoid any additional interactions between droplet oils and bacteria.

4.2 Detection of 1 CFU/mL of S. enterica – time dependence study:

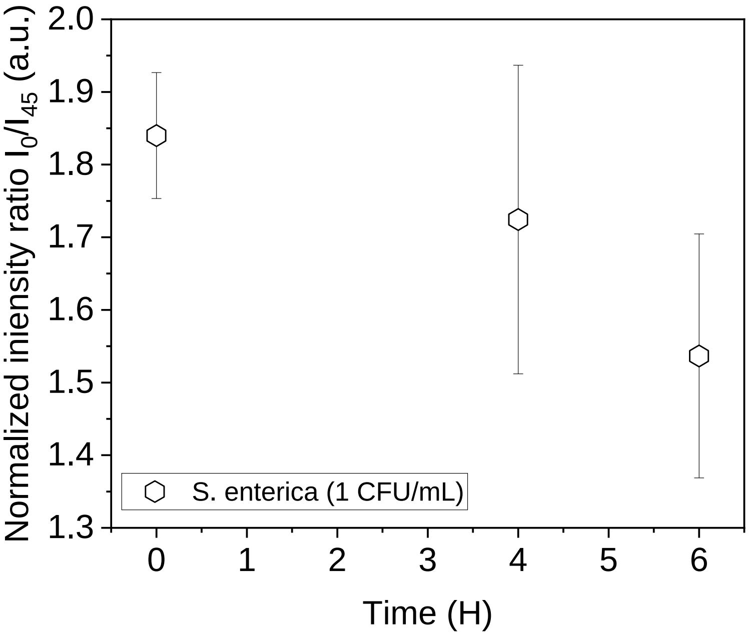

Fig. S28 Time-dependency studies on cleavage of surfactant **1** upon incubation with 1 CFU/mL of *S. enterica*. After 6 hours 1 CFU/mL is detectable.

4.3 Concentration dependence and theoretical limit of detection (T-LOD)

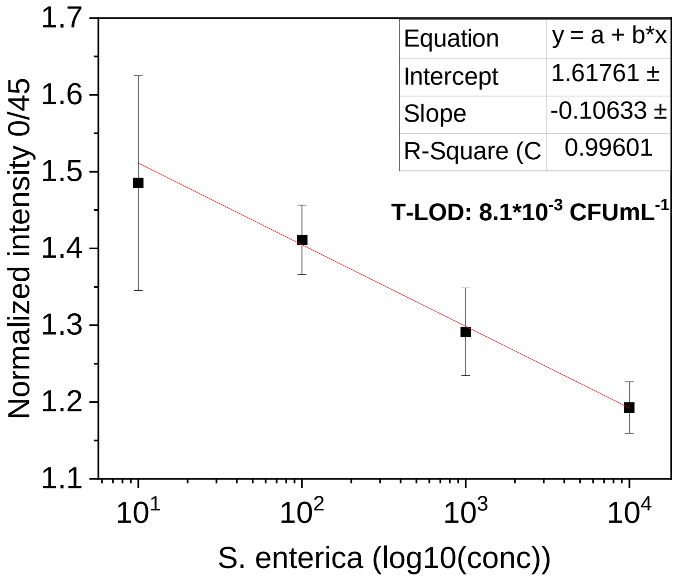

Fig. S29 T-LOD calculation for *S. enterica* concentration dependence tests:

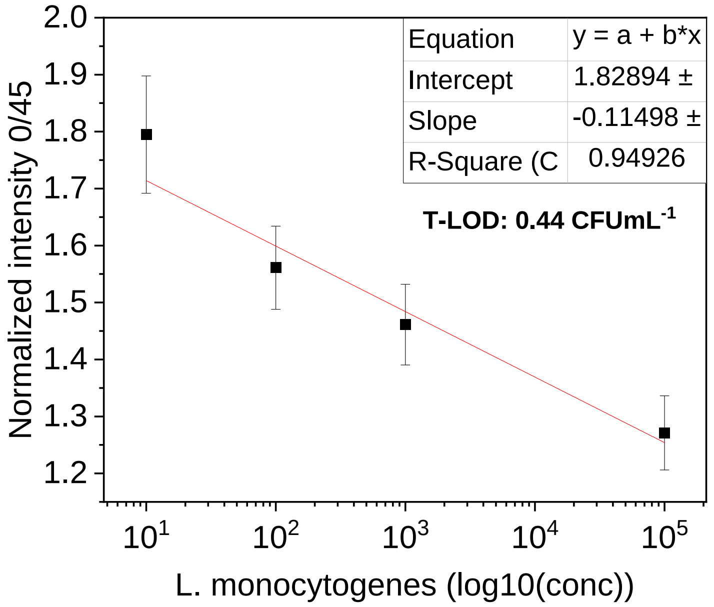

Fig. S30 T-LOD calculation for *L. monocytogenes* concentration dependence tests:

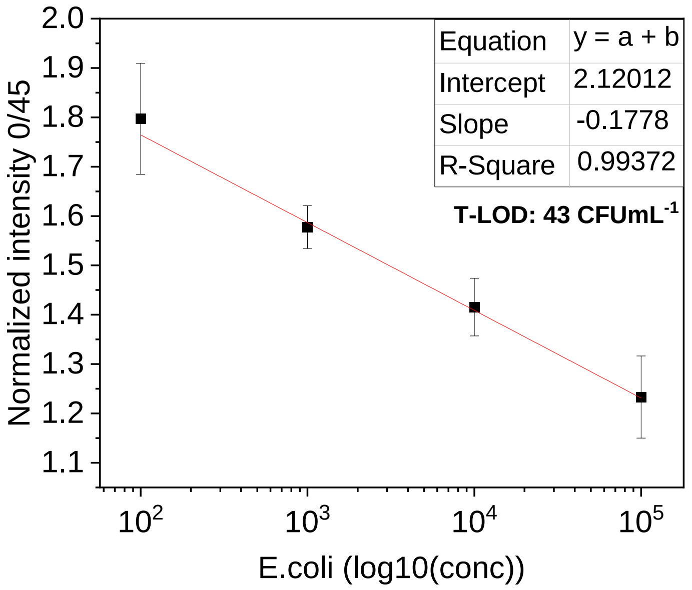

Fig. S31 T-LOD calculation for *E. coli* concentration dependence tests:

4.4 Cross tests:

S. enterica **-** Cross tests using emulsions stabilized with surfactant **2** and surfactant **3** were investigated. Dilution of bacterial broth solution was prepared in PBS yielding 10^2 CFU/ml, 100 µL was then added to 900 µL of surfactant 2 solution containing 300 µL of 1wt% of surfactant **2** – and 500 µL of 0.2wt% Zonyl and 100 µL of PBS buffer. Sample was then incubated for 2 hours. Analogous procedure was carried out for surfactant **3**: 100 µL of 10^5 CFU/ml of *L. monocytogenes* was added to 900 µL of surfactant **3** solution containing 400 µL of 1wt% Surfactant **3** – and 500 µL of 0.2wt% Zonyl. Samples were then incubated for 6 hours at 37°C. Subsequently, samples were placed on a heating stage along with separate vial containing 1:1 volume mixture of DEB with 2.5mM perylene and HFE7500. Vials were heated above the Tc of the oil mixture and 100 µL of oil mix was transferred into surfactant samples. Samples were then vortexed at 2100rpm for 10s ale left to cool down to room temperature. Changes in droplet morphology were compared with blank samples, where 100 µL of bacterial sample was replaced with 100 µL of PBS. Morphology changes were then recorded using a portable phone microscope and optical setup. All data points were measured in triplicates.

L. monocytogenes **-** Cross tests using emulsions stabilised with surfactant **1** and surfactant **3** were investigated. Dilution of bacterial broth solution was prepared in PBS yielding 10^3 CFU/ml, 100 µL was then added to 900 µL of surfactant 1 solution containing 300 µL of 1wt% of surfactant **1** – and 500 µL of 0.2wt% Zonyl and 100 µL of PBS buffer. Sample was then incubated for 6 hours. Analogous procedure was carried out for surfactant **3**: 100 µL of 10^5 CFU/ml of *L. monocytogenes* was added to 900 µL of surfactant 1 solution containing 400 µL of 1wt% Surfactant **3** – and 500 µL of 0.2wt% Zonyl. Samples were then incubated for 6 hours at 37°C. Subsequently, samples were placed on a heating stage along with separate vial containing 1:1 volume mixture of DEB with 2.5mM perylene and HFE7500. Vials were heated above the Tc of the oil mixture and 100 µL of oil mix was transferred into surfactant samples. Samples were then vortexed at 2100rpm for 10s ale left to cool down to room temperature. Changes in droplet morphology were compared with blank samples, where 100 µL of bacterial sample was replaced with 100 µL of PBS. Morphology changes were then recorded using a portable phone microscope and optical setup. All data points were measured in triplicates.

***E.coli* -** Cross tests using emulsions stabilised with surfactant **1** and surfactant **2** were investigated. Dilution of bacterial broth solution was prepared in PBS yielding 10^4 CFU/ml, 100 µL was then added to 900 µL of surfactant **1** solution containing 300 µL of 1wt% of surfactant **1** – and 500 µL of 0.2wt% Zonyl and 100 µL of PBS buffer. Sample was then incubated for 4 hours. Analogous procedure was carried out for surfactant **2**: 100 µL of 10^5 CFU/ml of *E.coli* was added to 900 µL of surfactant solution containing 300 µL of 1wt% of surfactant **2** – and 500 µL of 0.2wt% Zonyl and 100 µL of PBS buffer. Samples were then incubated for 6 hours at 37°C. Subsequently, samples were placed on a heating stage along with separate vial containing 1:1 volume mixture of DEB with 2.5mM perylene and HFE7500. Vials were heated above the Tc of the oil mixture and 100 µL of oil mix was transferred into surfactant samples. Samples were then vortexed at 2100rpm for 10s ale left to cool down to room temperature. Changes in droplet morphology were compared with blank samples, where 100 µL of bacterial sample was replaced with 100 µL of PBS. Morphology changes were then recorded using a portable phone microscope and optical setup. All data points were measured in triplicates.

4.5 Swab tests:

***S.enterica* - *S.enterica* -** 10^3 CFU/mL , 10^4 CFU/mL and 10^7 CFU/mL dilutions prepared from $8*10^8$ CFU/mL stock in TBS broth and diluted with PBS buffer. 100µL of each dilution was spilled onto a plate and wiped with an inoculation loop. The loop was then rinsed with 1mL of TBS growth medium. The same procedure was carried out with blank sample, where 100µL of PBS was spilled onto a plate, wiped with an inoculation loop and then rinsed with 1mL of TBS broth to ensure that plates are not contaminated. All samples were placed in the incubator for 2 hours at 37°C and 250 rpm. Subsequently, 100 µL of bacteria solution was transferred to 900 µL of surfactant **1** solution and further incubated at 37°C for 2 hours**.** Subsequently, samples were placed on a heating stage along with separate vial containing 1:1 volume mixture of DEB with 2.5mM perylene and HFE7500. Vials were heated above the Tc of the oil mixture and 100 µL of oil mix was transferred into surfactant samples. Samples were then vortexed at 2100rpm for 10s and left to cool down to room temperature. Changes in droplet morphology were compared with blank samples, where 100 µL of bacterial sample was replaced with 100 µL of PBS. Morphology changes were then recorded using a portable phone microscope and optical setup. All data points were measured in triplicates.

L. monocytogenes - 10^4 CFU/mL and 10^7 CFU/mL dilutions prepared from $8*10^8$ CFU/mL stock in BHI broth and diluted with PBS buffer - 100µL of diluted solution was spilled onto a plate and wiped with an inoculation loop. The loop was then rinsed with 1mL of BHI growth medium. The same procedure was carried out with blank sample, where 100µL of PBS was spilled onto a plate, wiped with an inoculation loop and then rinsed with 1mL of LB broth to ensure that plates are not contaminated. All samples were placed in the incubator for 2 hours at 37°C and 250 rpm. Subsequently, 100 µL of bacteria solution was transferred to 900µL surfactant **2** solution and further incubated at 37°C for 6 hours**.** Subsequently, samples were placed on a heating stage along with separate vial containing 1:1 volume mixture of DEB with 2.5mM perylene and HFE7500. Vials were heated above the Tc of the oil mixture and 100 µL of oil mix was transferred into surfactant samples. Samples were then vortexed at 2100rpm for 10s ale left to cool down to room temperature. Changes in droplet morphology were compared with blank samples, where 100 µL of bacterial sample was replaced with 100 µL of PBS.

Morphology changes were then recorded using a portable phone microscope and optical setup. All data points were measured in triplicates.

E.coli - 10^4 CFU/mL and 10^7 CFU/mL dilutions prepared from $8*10^8$ CFU/mL stock in LB broth and diluted with PBS buffer - 100µL of diluted solution was spilled onto a plate and wiped with an inoculation loop. The loop was then rinsed with 1mL of LB. The same procedure was carried out with blank sample, where 100µL of PBS was spilled onto a plate, wiped with an inoculation loop and then rinsed with 1mL of LB, to ensure that plates are not contaminated. All samples were placed in the incubator for 2 hours at 37°C and 250 rpm. Subsequently, 100 µL of bacteria solution was transferred to 900µL surfactant solution and further incubated at 37°C for 4 hours**.** Subsequently, samples were placed on a heating stage along with separate vial containing 1:1 volume mixture of DEB with 2.5mM perylene and HFE7500. Vials were heated above the Tc of the oil mixture and 100 µL of oil mix was transferred into surfactant samples. Samples were then vortexed at 2100rpm for 10s ale left to cool down to room temperature. Changes in droplet morphology were compared with blank samples, where 100 µL of bacterial sample was replaced with 100 µL of PBS. Morphology changes were then recorded using a portable phone microscope and optical setup. All data points were measured in triplicates.

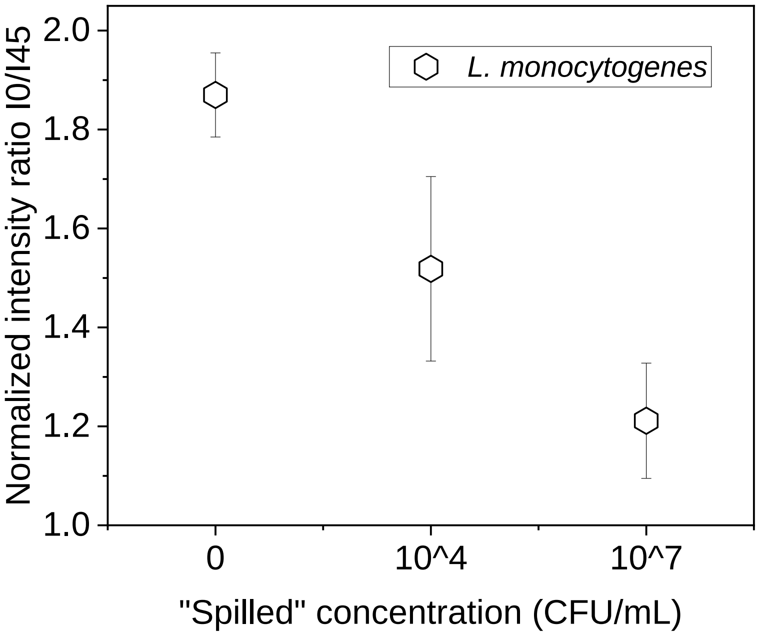

Fig. S32 Swab test results employing surfactant 3 to detect *L. monocytogenes*, where 0 corresponds to blank sample where only 100 µL of growth medium (broth) was added to the solution.

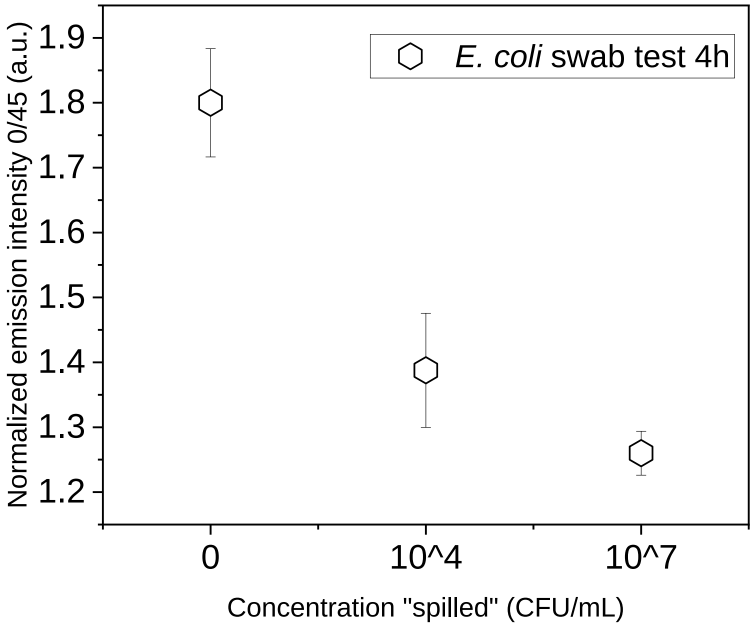

Fig. S33 Swab test results employing surfactant 3 to detect *E. coli*, where 0 corresponds to blank sample where only 100 µL of growth medium (broth) was added to the solution.

References:

[1] Stjerndahl, M.; Holmberg, K. Synthesis and chemical hydrolysis of surface-active esters. *J. Surf. Detergents* **2003**, *6*, 311-318.

[2] Schmidt-Lassen, J.; Lindhorst, T. K. Exploring the meaning of sugar configuration in a supramolecular environment: comparison of six octyl glycoside micelles by ITC and NMR spectroscopy. *MedChemComm* **2014**, *5*, 1218-1226.

[3] Zarzar, L. D.; Sresht, V.; Sletten, E. M.; Kalow, J. A.; Blankschtein, D.; Swager, T. Dynamically reconfigurable complex emulsions via tunable interfacial tensions. *Nature* **2015**, *518*, 520-524.

[4] Pavlovic, M.; Antonietti, M.; Schmidt, B. V. K. J.; Zeininger, L. Responsive Janus and Cerberus emulsions via temperature-induced phase separation in aqueous polymer mixtures. *J. Coll. Interface Sci.* **2020**, *575*, 88-95.

[5] Primiceri, E.; Chiriacò, M. S.; de Feo, F; Santovito, E.; Fusco, V.; Maruccio, G. A multipurpose biochip for food pathogen detection. *Anal. Meth.* **2016**, *8*, 3055-3060.

[6] Wang, L.; Regina, A.; Butardo Jr., V. M.; Kosar-Hashemi, B.; Larroque, O.; Kahler, C. M.; Wise, M. J. Influence of *in situ* progressive N-terminal is still controversial truncation of glycogen branching enzyme in *Escherichia coli* DH5α on glycogen structure, accumulation, and bacterial viability. *BMC Microbiology* **2015**, *15*, 96.

[7] Djalali, S.; Frank, B. D.; Zeininger, L. Responsive drop method: quantitative in situ determination of surfactant effectiveness using reconfigurable Janus emulsions. *Soft Matter* **2020**, *16*, 10419-10424.
